# Molecular regulation of conditioning film formation and quorum quenching in sulfate reducing bacteria

**DOI:** 10.3389/fmicb.2022.1008536

**Published:** 2022-10-31

**Authors:** Dheeraj Raya, Aritree Shreya, Anil Kumar, Shiv Kumar Giri, David R. Salem, Etienne Z. Gnimpieba, Venkataramana Gadhamshetty, Saurabh Sudha Dhiman

**Affiliations:** ^1^Department of Civil and Environmental Engineering, South Dakota Mines, Rapid City, SD, United States; ^2^2DBEST Research Center, South Dakota Mines, Rapid City, SD, United States; ^3^Centre for Medical Biotechnology, Maharshi Dayanand University, Rohtak, Haryana, India; ^4^Department of Biotechnology, Maharaja Agrasen University, Baddi, Himachal Pradesh, India; ^5^Chemical and Biological Engineering, South Dakota Mines, Rapid City, SD, United States; ^6^Department of Biomedical Engineering, University of South Dakota, Vermillion, SD, United States; ^7^Department of Chemistry, Biology and Health Sciences, South Dakota Mines, Rapid City, SD, United States

**Keywords:** copper toxicity, machine learning, quorum sensing, conditioning protein, homology modeling, metabolic flux

## Abstract

Sensing surface topography, an upsurge of signaling biomolecules, and upholding cellular homeostasis are the rate-limiting spatio-temporal events in microbial attachment and biofilm formation. Initially, a set of highly specialized proteins, *viz*. conditioning protein, directs the irreversible attachment of the microbes. Later signaling molecules, *viz*. autoinducer, take over the cellular communication phenomenon, resulting in a mature microbial biofilm. The mandatory release of conditioning proteins and autoinducers corroborated the existence of two independent mechanisms operating sequentially for biofilm development. However, both these mechanisms are significantly affected by the availability of the cofactor, e.g., Copper (Cu). Generally, the Cu concentration beyond threshold levels is detrimental to the anaerobes except for a few species of sulfate-reducing bacteria (SRB). Remarkably SRB has developed intricate ways to resist and thrive in the presence of Cu by activating numerous genes responsible for modifying the presence of more toxic Cu(I) to Cu(II) within the periplasm, followed by their export through the outer membrane. Therefore, the determinants of Cu toxicity, sequestration, and transportation are reconnoitered for their contribution towards microbial adaptations and biofilm formation. The mechanistic details revealing Cu as a quorum quencher (QQ) are provided in addition to the three pathways involved in the dissolution of cellular communications. This review articulates the Machine Learning based data curing and data processing for designing novel anti-biofilm peptides and for an in-depth understanding of QQ mechanisms. A pioneering data set has been mined and presented on the functional properties of the QQ homolog in *Oleidesulfovibrio alaskensis* G20 and residues regulating the multicopper oxidase properties in SRB.

## Introduction

Sulfate reducing bacteria (SRB) can be broadly categorized as mesophilic microbes, with the exception of thermophilic strains isolated from the oil and gas industry drainages, displaying autotrophic, litho-autotrophic, or heterotrophic respiration types under anaerobiosis (Mayilraj et al., 2009; Fichtel et al., 2012; Jia et al., 2018). Numerous species of sulfate reducing bacteria *viz*. *Desulfovibrio alaskensis* (Wikieł et al., 2014), *Desulfovibrio vulgaris* (Ueki and Lovley, 2022), *Desulfovibrio brasiliensis* (Warthmann et al., 2005), and *Desulfovibrio ferrophilus* (Chatterjee et al., 2021) are reported for their biofouling, biofilm forming, and microbially induced corrosion (MIC) properties. The impact of the MIC will be more profound as it causes putrefaction of the metal pipelines, which may result in an oil spillage scenario by damaging supply pipelines. The detrimental impact of MIC is led by the biofilms originating from the microcosmic heterogenic microbial aggregations. This localized microbial aggregation is controlled by the surface properties, including microbe-metal interactions leading to the conditioning film development. Reports validate the conditioning film’s development within the first few minutes of microbial interactions with the surface. The nature of the conditioning films differs significantly depending on the natural landscapes, microcosmic environment, *viz*. metal type, nutrient availability, hydrophobicity, etc. (Lorite et al., 2011). Conversely, the growing conditioning film also changes the surface properties, e.g., hydrophobicity, hydrophilicity, roughness, and charge (Arnaouteli et al., 2016). Reports describe the complex proteinaceous nature (Fong and Yildiz, 2015) of the conditioning films rich in exopolysaccharides, glycolipids, and other microbial secretions. Fibrinogen and fibronectins are the documented conditioning proteins (CP) dominating the pathogenic conditioning film on human implants (Nandakumar et al., 2013; Souza et al., 2015; Khatoon et al., 2018). Human proteins such as albumin, vitronectin, and collagens are adsorbed on the surface of implants, making them suitable for bacterial attachment. Despite the complexity in the process of adsorption, the adsorption of proteins on the surface would increase with time, at least until the adsorbed proteins approach monolayer coating. Over time, without cellular interaction, absorbed layers are displaced by proteins such as kininogen with higher surface affinity. However, the family, structure, and properties of the homologous counterparts in biofilms growing under natural landscapes, e.g., hot springs, deep biosphere, water channels, and rhizospheres, are unexplored and undocumented.

In addition to surface properties, micronutrient availability also influences SRB colonization. Microbes widely use copper (Cu) as a micronutrient and cofactor in numerous redox enzymes, e.g., cytochrome oxidase, transcription regulators, etc. At elevated concentrations, Cu is inhibitory toward SRB growth; hence, preferred for industrial pipelines, offshore installations, etc., where microbial colonization is a challenge. The presence of Cu restricts colonization by facilitating reactive oxygen formation or replacing Fe^2+^ in the iron–sulfur (Fe-S) cluster of the critical enzymes. Contrary to the discernment of Cu as a quorum quencher (QQ), biofilms have been observed on Cu and nickel surfaces due to novel homeostasis mechanisms developed by a few species of SRB (Rodrigues et al., 2008). Despite the reporting on Cu as a QQ, relevant mechanistic details are underexplored in the case of SRB.

**Figure 1:**
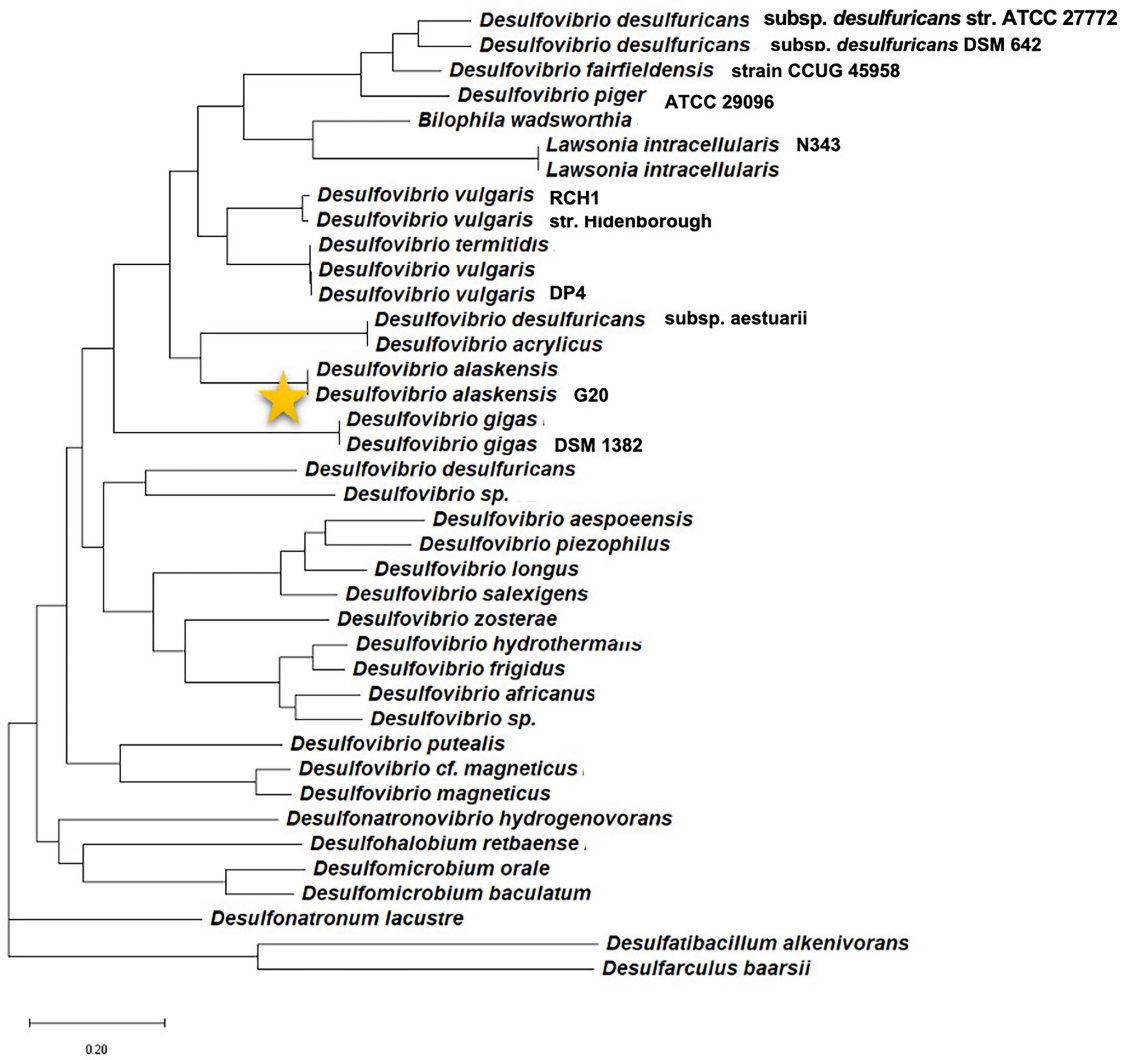
Phylogenetic position of the model organism *Desulfovibrio alaskensis* G20 among different sulfate reducing bacteria.

In the current scenario, a comprehensive review detailing initial cell attachment and cellular communications is well due as reports are available detailing biochemical events resulting in a mature SRB’s biofilm. With the broad-spectrum applicability of the Machine Learning (ML) platform, enormous opportunities have arisen in finding efficient QQ molecules and understanding common trends from the available scientific data. Therefore, relatively underexplored *Oleidesulfovibrio alaskensis* G20 has been selected as a model SRB to decode the roles of the multicopper oxidases and lactonases proteins actively involved in the colonization and communication stages. The phylogenetic information of the *D*. *alaskensis* G20 is given in Figure 1, and several unique features of the SRB are given in Table 1. The potentials of the ML platform have been investigated for facilitating the experimental validation of signaling molecules and designing anti-biofilm peptides. Given the ecological and industrial significance of the SRB biofilms, an assessment of literature from the past 15 years till June 2022 has been carried out to investigate the roles of (i) conditioning films and their constituents in microbial colonization, (ii) SRB adaptations towards elevated levels of copper, and (iii) quorum quenching mechanisms and role of ML in identifying novel quenching peptides for SRB.

**Table 1:** Overview of gene expression profiling and adaptations displayed by sulfur reducing bacteria in response to Cu-toxicity.

Expression level	Microbe	Genes	Cellular functionality	References
Downregulated	*Desulfovibrio vulgaris* strain Hildenborough	DVU0307, DVU1443, and DVU1444	Cell motility	Copper (2018)
		DVU3132	Lactate oxidation	
		*dcrH*, *DVU0170*, *CheW-1*, DVU0591, and DVU0700	Chemotaxis*	
	*Oleidesulfovibrio alaskensis* G20	Dde_0356, Dde_ 2,958	Cell motility	Hao et al. (1994)
		Dde_3239	Lactate oxidation	
		*CheD*, *CheW*, *CheC*, *Chez*	Chemotaxis*	
		*feoA*, and Dde_2669	Inorganic ion transporter	
Upregulated	*Desulfovibrio vulgaris* strain Hildenborough	DVU2571, DVU2572, and DVU2574	Inorganic ion transporter	Copper (2018)
		DVU1257, *hup-2*	DNA replication	
	*Oleidesulfovibrio alaskensis* G20	Dde_2324	DNA replication	Hao et al. (1994)

* Includes methyl-accepting chemotaxis protein and cytosolic chemotaxis protein only.

## Conditioning film

The majority of microbial colonization, their evolutionary developments, and two-thirds of human infections involve conditioning film formation (Nadell et al., 2008). A conditioning film is a layer of proteinous compounds and organic biomolecules facilitating the initial irreversible attachment of the planktonic microbial cells, which consequently results in a mature biofilm formation. A conditioning film appears prior to microbial attachment and is made up of organic compounds bound to the external surface through van der Waals interactions. Deposition of biomolecules facilitates the planktonic cell attachment irreversibly *via* modulating the surface properties (Zobell, 1943; Lorite et al., 2011). For example, an increase in surface roughness and hydrophobic characteristics is often observed prior to developing a mature biofilm (Oh et al., 2009). In the case of Xylella fastidiosa, the existence of conditioning film was reported within 2 h of inoculation on terrestrial objects, and a gradual increase (up to 100 nm) in thickness was observed within a few days (Lorite et al., 2011).

Compared to terrestrial conditions, a quick accumulation of conditioning film components was observed for submerged surfaces in the marine environment (Friedlander et al., 2013). While the biomolecules regulating the later stages of the microbial attachments, e.g., microcolony formation, biofilm maturation, and detachment (also termed dispersal), are rationally characterized (Kostakioti et al., 2013; Muhammad et al., 2020), little is known about the proteins formulating the conditioning film under natural landscapes. No specific details on class, type, structural and functional properties of the proteins regulating microbial settling in natural landscapes are listed. In contrast, numerous reports are available on the proteins serving as conditioning film components (known as conditioning protein) on human implants under controlled conditions.

Given the information on microbial biofilms in natural caves, industrial pipes, rhizosphere, and other natural landscapes (Harjai and Sabharwal, 2017), a similar conditioning film formulation mechanism mediated by the conditioning proteins is hypothesized. It is hypothesized that proteinous components are released only during the early developmental stages (milliseconds to first few minutes), and their secretion is independent of the characteristic compounds, e.g., exopolysaccharides, released at the later stages of the biofilm. The initial sensing of the surface properties in search of carbon and energy sources is mediated by the ions and solutes available in the microcosmic environments. The initial sensing (within micrometer range) of the carbon source triggers the release of the surface receptors by the microbes, followed by the highly specific conditioning proteins to develop a protein-receptor complex, facilitating the microbial attachment. This irreversible attachment of the planktonic cells initiates the release of the signaling molecules, another specific class of biomolecules termed as autoinducers, and establishes the phenomenon of quorum sensing. Biochemical events followed by the QS establishment are also typical for the natural landscapes reported for other microbial species in other environments. For example, specific proteins, *viz*. cytochrome-C oxidase (chemotaxis transducer) and MexH (regulatory subunit), are reported only for the final stages of the biofilm. Mutated cytochrome-C oxidase and MexH did not confer any defect in the conditioning film formation. Conversely, their mutagenesis resulted in a pause in the biofilm development at the cell cluster stage. These observations strengthen the hypothesis that two independent mechanisms operate sequentially for developing a microbial biofilm. The early-stage mechanism is related to the development of conditioning film *via* the synthesis and release of conditioning proteins. The mechanism active at the later stages mediated by the signaling molecules is termed quorum sensing (QS) and is responsible for the mature biofilm development. Given the significance of the initial microbial settling, an in-depth understanding of the conditioning film, its development, and constituent conditioning proteins will transform the sulfate reducing bacteria biofilm research.

### Conditioning film components

Conditioning film’s composition varies rendering to the Spatio-temporal microcosmic conditions. Therefore, the rate-limiting step establishing the preliminary microbial attachments differs significantly. Numerous chromatographic, fluorescence-based imaging and crystallographic methods were deployed to validate the presence of proteinous molecules, glycolipids, carbohydrates, nucleic acids, polysaccharides, aromatic amino acids, and humic acid in the conditioning film (Bakker et al., 2004). The majority of proteinous compounds for SRB are uncharacterized in the case of biofilm growing under natural landscapes. However, the X-ray photoelectron analysis confirmed the adsorption of proteinous compounds as pioneering biomolecules for triggering the initial attachment, as validated through increased oxygen and nitrogen concentrations on the surface over time. In contrast, in the marine environment, the presence of aldoses such as rhamnose, arabinose, xylose, and glucose, uronic acid was confirmed for conditioning film immersed in seawater (Garg et al., 2009).

All characterized components exhibited affinity toward the cell surface, resulting in the adhesive bond formation and thus improved adhesion efficiency (Berne et al., 2018). Mainly the phosphate groups of the denatured biomolecules and modified proteinous compounds were involved in bond formation with the functional groups exposed on the surface of the microbial cell (Lorite et al., 2011). For example, Friedlander et al. (2013) studied the ability of *Escherichia coli* and SRB to precondition the surface and subsequent impact on the surface wettability characteristics. It was determined that bacterial modification of the surface was the predominant contributor to change in surface wettability and bacterial adhesion (Friedlander et al., 2013). Likewise, the type-IV pilin protein (*PilA*) mutant of *Pseudomonas aerugonisa* PAO1 exhibited a decreased rate of microcolony formation due to the unavailability of the surface functional groups for attachment (Zhao et al., 2013). In the case of another conditioning film forming facultative anaerobe, *Pseudomonas aerugonisa*, a trail of polysaccharide synthesis locus (Psl) type exopolysaccharides was deposited on the unconditioned clean surface (Zhao et al., 2013).

#### Conditioning proteins

Limited microbial proteins have been identified and reported for a controlled environment (e.g., biomedical, pathogenesis research) serving as the conditioning proteins (CP). The primary reasoning for limited information on CP is due to their isolation from a highly specialized research area of human implants and allied pathogenesis (Foster, 2020). A controlled, nutrient-rich, fluidic microenvironment maintained by the human body’s homeostasis makes it feasible to extract the quantifiable CPs. The majority of the characterized proteins are coagulable, displaying dimeric structure and bivalent properties. What confers to the adsorption mechanism of the CPs structure to surface highly depends on electrostatic interactions between protein and surface and results in changes of conformational entropy of protein. Hagiwara et al. (2009) showed that after initial surface adsorption of β-lactoglobulin through van der Waals interaction, some acidic residues dissociate, strengthening the protein-surface binding (Hagiwara et al., 2009). Initially, proteins adapt to an orientation that maximizes the favorable surface-protein interaction resulting from the electrostatic attraction. However, with an increase in the concentration of adsorbed protein on the surface, this orientation becomes unfavorable, leading toward an increased desorption rate (Rabe et al., 2011). A detailed mechanism of protein adsorption on a solid surface, along with the mathematical concepts and model description was discussed (Rabe et al., 2011). As the current review is focused on the SRB in a natural ecosystem, detailed descriptions of CPs from biomedical research directions are beyond the scope of the article.

Given the microbial growth kinetics, a mechanistic similarity is expected for the SRB colonization under natural landscapes. Therefore, portentous molecules of SRB exhibiting similar structural and functional characteristics are expected to be involved in the formulation of the conditioning film. A few reports on microbes, including *Pseudomonas* spp., and *Xanthomonas* spp. highlight the presence of amide I, amide II, aromatics, and ester peaks corresponding to the conditioning proteins deposited on the iron heaps after 72 h under offshore conditions (Compère et al., 2001). Likewise, 12 natural amino acids and one non-protein amino acid were extracted from the conditioning film of marine culture, *Bacillus* spp., and *Pseudomonas* spp., growing on the glass coverslip exposed to the seawater (Bhosle et al., 2005; Jain and Bhosle, 2009).

### Conditioning film in the marine environment

In the marine environment, conditioning film development may proceed *via* multiple convergent signaling pathways influenced by dynamic and uncertain environmental conditions (Garg et al., 2009). One such pathway involved triggering of chemotaxis movement toward deposited organic compounds. In general, planktonic cells feed upon these organic compounds coming from the marine flora and fauna.

Adsorption of organic compounds is reported within a few hours of immersing an external surface in the marine environment and is later confirmed by analyzing total organic carbon (TOC; Loeb and Neihof, 1975; Jain and Bhosle, 2009). Similar results were corroborated by Bakker et al. (2004), in which increased surface nitrogen was observed on polyurethane coating within 1 h after immersing it in seawater. Similar observations are reported for the total hydrolyzable amino acids, increasing total organic carbon and nitrogen (Bakker et al., 2003, 2004). Elemental analysis of the marine conditioning film revealed a compositional frameshift from degraded matter (in the initial phase) to the non-degraded matter (in the maturation phase). These studies suggest that the initial reduction in the total TOC content of a conditioning film might be due to the consumption of nutrients by the growing microbial cells.

### Conditioning film in the natural ecosystem

Degradation of environmental pollutants, organic matter, and maintenance of different organic cycles (e.g., C and N) require cumulative effort from microbes, including SRB exhibiting various metabolic activities. Bacterial conditioning film has been found ubiquitously even in extreme natural environments such as acid mine drainage (Kalin et al., 2018), ice lakes in Antarctica (Smith et al., 2016), and thermal springs (Bhagwat et al., 2021) due to their metabolic plasticity. Proteobacteria, Bacteroidetes, and Cyanobacteria dominate biofilm forming bacteria in the aquatic ecosystem. Different proteobacteria take advantage of living or decaying plants, diatom aggregates, and humic substances (Newton et al., 2011) in comparison to refractory organic materials preferred by Bacteroidetes and cyanobacteria to formulate the conditioning film for initial attachment. Hwang et al. (2013) studied the effect of alginate, humic acid, fulvic acid, and bovine serum albumin and their impact on bacterial adhesion to conditioning film formation in aquatic environments. This study summarized the favorable impact of low concentrations (1–10 mM) of natural substrates over high concentrations (~100 mM) in developing the conditioning film in the aquatic ecosystem. Miao et al. (2021) have found chemo-heterotrophy as the most dominant biogeochemical cycle in the microbial world involved in conditioning film development on wood surfaces. Intact and fragmented conditioning films are reported for the *Actinomycetes*, *Pseudomonas* sp., and *Rhodococcus* sp. on rock surfaces (Gorbushina, 2007). The conditioning film formation within the deep rock fractures has also been corroborated by Schäfer et al. (2015) with the presence of fucose, phenylalanine, and other exopolysaccharides forming amino sugars.

### Conditioning film in industrial system

The resilient microcosmic environment developed due to the conditioning film helps maintain the microbial growth by nutrients entrapment within the biofilm matrix (Gilbert et al., 1990). Conditioning film and subsequent mature microbial biofilm provide a heterogenous, resilient environment to the growing cell line against the odd of disinfectants, biocides, antibiotics, etc., draining through the industrial pipelines. Bacterial species *viz*. *Bacillus cereus*, *E*. *coli*, *Listeria monocytogenes*, *Salmonella enterica*, and *Staphylococcus aureus* are the predominating species responsible for initial colonization and biofilm formation in the food industry. It also raises health concerns from dairy products and ready-to-eat foods. The conditioning film formation on fish products is governed by organic molecules such as troponin, tropomyosin, myosin, and apolipoprotein, whereas α-casein, β-casein, κ-casein, and α-lactalbumin help condition film formation in dairy products (Whitehead and Verran, 2015). Tryptone soya broth has facilitated conditioning film formation on vacuum-packed meat and chicken. Sulfate reducing bacteria, e.g., *Desulfobulus* spp. *Desulfobacterium autotrophicum*, and *Desulfotalea psychrophila* are found in the water circulation system of the pulp and paper industry, where they cause biofilm formation and microbial corrosion aided by the presence of a high concentration of chlorine (Hussain et al., 2016; Virpiranta et al., 2019). The presence of organic macromolecules such as polysaccharides and polypeptides in drinking water has been proved to proliferate conditioning film formation on hydrophobic surfaces used as liners for drinking water systems (Francius et al., 2017). SRB attachment can also be seen in other industries, such as chemical processing, cooling water, and air conditioning, where they cause different environmental and health concerns. It has been observed that an increase in surface resistance increases the conditioning film formation and subsequent biofouling in the process industry (Kukulka et al., 2004).

## Antagonism between copper ions and microbial colonization

Sulfate reducing bacteria is a heterotrophic anaerobic organism capable of utilizing several organic compounds, including aromatic hydrocarbons, and generates sulfide by reducing sulfate and copper amendment effects in bacterial communities enriched from sediments exposed to copper mining residues. Due to its ability of carbon mineralization in the sulfate-rich natural environment, SRB accounts for ~50% of TOC oxidation in ecosystems (Pérez-Jiménez et al., 2001). However, carbon mineralization relies on the synergistic behavior of enzymatic cocktails, including cytochrome oxidases, metal dehydrogenases, etc., which in particular are affected by heavy metals, e.g., copper (Cu). For instance, metabolic activity, primarily sulfate reduction by SRB, decreased considerably with the increasing concentrations of heavy metals, specifically Cu (Zampieri et al., 2022). Hence, the antimicrobial characteristics of the Cu garnered tremendous attention for developing the marine infrastructure.

Several studies have demonstrated the toxic nature of Cu toward the SRB (Zampieri et al., 2022). Copper metal completely inhibits SRB mixed culture at a concentration ~ 4 mg/L (Hao et al., 1994), and ~50% reduction in total cell protein was reported at a concentration of 15 μM of Cu(II) for *Oleidesulfovibrio alaskensis* G20 (Tripathi et al., 2022). In the case of *O*. *alaskensis* G20 growth rate decreased from 0.17 to 0.10 h^−1^ when the concentration of Cu(II) was increased upto 15 μM. For *Citrobacter* sp. strain DBM, sulfate reduction activity ceased at a concentration of 0.6 mM (Qiu et al., 2009), whereas that of *Desulfovibrio vulgaris* was at 0.06 mM (Cabrera et al., 2006). The antibacterial characteristics are attributed to release of Cu ions from metal surfaces possessing high toxicity (Nan et al., 2015) and as well as killing of bacteria by metallic copper surfaces by a phenomenon called as contact killing.

**Figure 2:**
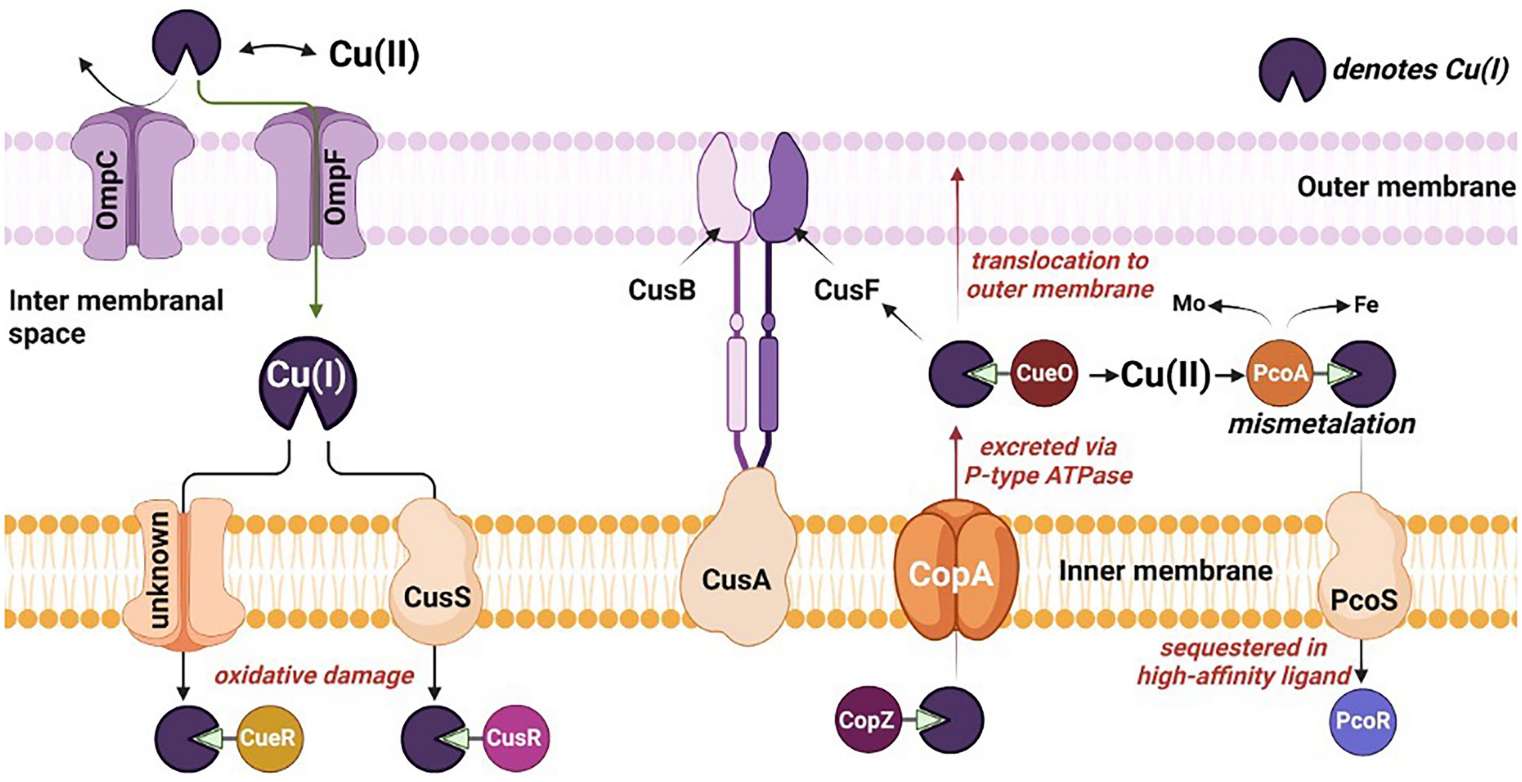
Mechanistic overview of the toxicity, sequestration, and translocation of copper in sulfate reducing bacteria. Omp, outermembrane protein; Cus, copper exporter protein; Cop, Cu^+^-ATPase protein; and Pco, periplasmic copper binding protein.

Remarkably, numerous microbial strains, e.g., *Desulfovibrio* sp. A2, *Desulfosporosinus* sp. I2 have developed resilient characteristics towards inhibitory compounds, including Cu-metal. Prolonged exposure to Cu has developed the resistance toward metal toxicity (Hao et al., 1994) and eventually resulted in the adherence of the SRB to the Cu surface resulting in biofilm formation. The redox activity of SRB leads to formation of biogenic hydrogen sulfide. Hydrogen sulfide is a powerful reactant and reacts with metal surfaces accelerating the process of MIC. A thick biofilm up to 15 μm is reported for *D*. *alaskensis* G20 on the Cu surface within 27 days of exposure (Chilkoor et al., 2020). The potential mechanism of Cu toxicity and Cu handling machinery evolved in the bacterial cells has been shown in Figure 2 and discussed in the following sections.

### Copper toxicity mechanism

Numerous reports decipher the underlying mechanisms of Cu-toxicity observed among different microbial genera. The universally accepted mechanism signifies the role of oxygen atom in Cu-precipitation and hydroxyl radical production to mediate a Fenton-type reaction, resulting in oxidative damage to macromolecules, e.g., proteins, lipids, and DNA (Ladomersky and Petris, 2015). These reactive hydroxyl radicals formed are usually amplified *via* Haber-Weiss reactions to produce reactive oxygen species (ROS) such as O_2_^−^ and H_2_O_2_, amplifying the oxidative stress on the cells. Increasing the concentration of Cu(II) leads to overproduction of ROS, resulting in decreased sulfate utilization in *D*. *vulgaris* Hildenborough. However, these ROS have a short half-life and are detrimental to macromolecules within Cu′s vicinity (Ladomersky and Petris, 2015). It has also been reported that Cu leads to depletion of glutathione, which acts as a protection against heavy metal toxicity. It was proposed that glutathione binds with Cu and facilitates delivery to the Fe-S cluster of essential enzymes (Copper, 2018).

In the absence of O_2_, displacement of Fe by Cu in Fe-S cluster and formation of thiolate bond by Cu have been reported. The Fe-S cluster is organometallic assemblies of Fe and S responsible for biological electron transfer, which serves as Fe and S repositories, and plays a role in genomic stability and nucleic acid metabolism. Its inactivation leads to border impacts such as respiration on physiological functions in the SRB. Ferrous iron transporter (Fur) regulon, the primary regulator of Fe-uptake and storage, like ferrous transport fusion *feoAB proteins* were highly induced in *D*. *vulgaris* Hildenborough, suggesting Cu has resulted in imbalance intracellular Fe metabolism *via* disturbance of Fe-S cluster. Typically, a Fur regulon is activated when the Fe is depleted and induces transcription of genes associated with Fe uptake and storage. In the case of *D*. *alaskesnsis* G20, the Fur transporter system was downregulated when exposed to a higher concentration of Cu.

Repressed energy conversion is a commonly observed phenomenon in SRB during Cu stress. A total of five genes (DVU3132, DVU3133, DVU2091, DVU2348, and DVU1081) in *D*. *vulgaris* Hildenborough (Chen et al., 2019) and lactate dehydrogenase (Dde_3239) in *D*. *alaskensis* G20 (Tripathi et al., 2022) involved in lactate oxidation were downregulated. Furthermore, the gene encoding for the 30 and 50s ribosomal protein was downregulated by ~90% in *D*. *alaskensis* G20. Cells respond to this energy crisis by downregulating the genes responsible for cell motility and chemotaxis, an energy-intensive mechanism. For example, genes governing flagella development (e.g., *flgK*, *flgD*, and *flgE*), flagella dependent motility, and methyl-accepting chemotaxis protein, were downregulated in *D*. *vulgaris* Hildenborough.

### Copper tolerance mechanisms

Three central molecular mechanisms have been developed by different SRB to counter metal toxicity. Precise regulation of the (i) enzymatic detoxification/reduction of Cu, (ii) energy-dependent efflux of Cu ions, and (iii) Cu sequestration are commonly reported mechanisms in a variety of sulfate reducing bacteria (Table 1). Copper influences an array of genotypic and phenotypic characteristics in SRB. It is required as a cofactor for essential microbial enzymes (Ladomersky and Petris, 2015), modulating the strength of the signaling molecules involved in QS (Grubman and White, 2014) and triggering stress-response mechanisms due to cellular toxicity (Baker et al., 2010). In-depth mechanistic details are available on the detrimental impacts of Cu on total cell protein, lag times, and specific growth rates (Sani et al., 2001). Sulfate reducing bacteria has developed an intriguing precipitation mechanism through biogenic sulfides to handle Cu-toxicity (Gramp et al., 2006). Conversely, limited reports are also available on the complete inhibition of microbial growth in excessive Cu-conc (Sani et al., 2001). Improved biofilm formation was observed for Corteen steel containing 2.25% (wt.) of Cu. Conversely, Ce addition to Cu bearing 2,205 duplex stainless steel reduces biofilm formation by modifying the membranal peptidoglycan controlling the cellular permeability. Considering the unexplored molecular and biochemical properties of SRB affected by the Ce, the compiled information is restricted to the Cu stress.

#### Enzymatic detoxification

Attainment of resilient characteristics against Cu-metal ions involves precision guided detoxification mechanisms mediated by the transmembrane protein. Such specialized proteins facilitate the conversion of the toxic Cu(I) into less toxic Cu(II) within the periplasm and limit Cu(I) from entering the cytoplasm (Hyre et al., 2021). Reports deciphering the role of proteins, e.g., membrane-associated multicopper oxidase (MmcO) in the *M*. *tuberculosis* and Cu(I) oxidizing multicopper oxidase (CueO) encoded by Cu efflux system (*cue*) in *E*. *coli* are available for conferring the copper tolerance by acting as binding or translating agents. Likewise, the role of a periplasmic multicopper oxidase, e.g., DA2_CueO protein, is reported for *Desulfovibrio* sp. A2 confers copper tolerance ~40 mM and encodes for multicopper oxidase with metal ion resistance role and enhanced copper tolerance (Mancini et al., 2017). Similarly, *Desulfosporosinus* sp. I2 confers copper tolerance ~142 mM and has several genes associated with P-type ATPase, resistance-nodulation-cell division transporters, and cation diffusion facilitators.

**Figure 3:**
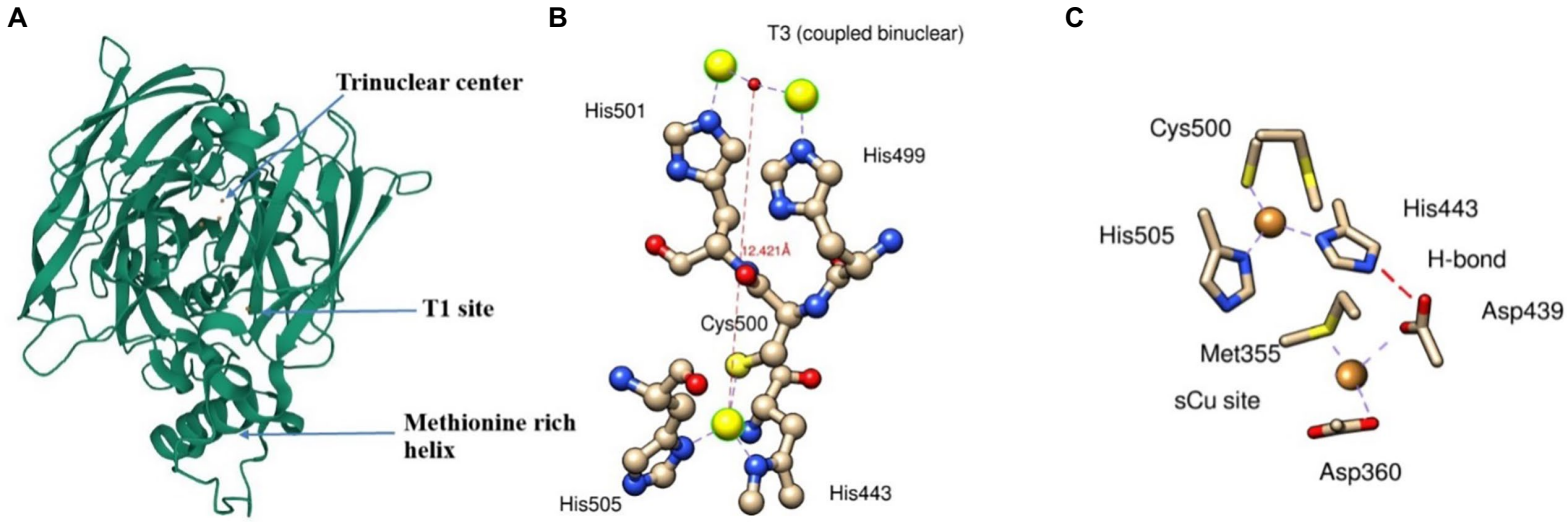
A ribbon diagram of CueO (PDB: 3OD3) showing **(A)** Trinuclear site, T1 site, and methionine rich helix, **(B)** interaction of T1 and T3 sites with its respective amino acids residue*, and **(C)** electron transfer from sCu into T1 site between Asp^439^, sCu, and His^443^. *Interaction of T3 site with T2 is not shown.

Cu(I) oxidizing multicopper oxidase is expressed and folded in the cytoplasm and exported to the periplasm by the twin-arginine motif *via* the twin-arginine translocation (Tat) pathway to produce folded proteins in the periplasmic space (Stolle et al., 2016). Three different coppers centers designated as type 1(T1), type 2 (T2), and type 3 (T3) are responsible for the redox reaction catalyzed by multicopper oxidase (MCO). T1 copper center (intense blue color typical of MCO) shows strong adsorption at 600 nm in UV visible spectra, T2 copper central, and T3 (coupled binuclear) shows absorbance at 330 nm (Singh et al., 2011; Kaur et al., 2019). T2 and T3 center in conjugation makes trinuclear center (Figure 3). T1 sites accept four-electron during substrate oxidation and transfer to the coupled binuclear cluster (T3) over 12 Å *via* Cys-His-His amino acid residue (Figure 3B). The transferred electron is used to reduce the oxygen in the water. The first two electrons involve the conversion of dioxygen to peroxide intermediate, and the second two-electron is used to reduce the intermediate to two water molecules (Kaur et al., 2019).

The methionine-rich sequence in MCO has several active sites for binding Cu ions and performs cuprous oxidase activity. The N-terminal portion of methionine-rich insert (residues 355–379) contains Met^355^ and Asp^360^, which plays a crucial role as substrate copper (sCu) binding site and have several sites for binding with Cu(I). Cu(I) bound to sCu sites in methionine-rich insert undergoes oxidation to release an electron. These electrons transfer to the T1 site *via* a hydrogen bond between Asp^439^, sCu, and His^443^ and ultimately into T2/T3 cluster (Figure 3c). However, the replacement of Cu(I) into this sCu site is yet to be determined.

#### Energy dependent efflux

Copper homeostasis has been well studied and characterized only in a few Gram-negative bacteria, e.g., *M*. *trichosporium*, *Synechocystis* PCC689. In, *Desulfosporosinus* sp. OT genome contains two CopA (Cu-exporting ATPases), one each subunit of CopU (regulates the operon) and CopZ (Cu chaperone). In *D*. *Vulgaris* Hildenborough, P-type ATPase (gene ID: DVU2800, DVU3332) responsible for controlling and translocating the heavy metal ion in periplasm and cytoplasm showed upregulated expression. This mechanism of Cu-efflux adapted by SRB shows the active mechanism for exporting the copper through the periplasm to the extracellular environment. Similarly, in *Lactococcus lactis*, a *copRZA* operon is the core element of Cu resistance where CopR encodes for copper-inducible repressor, and CopZ acts as a Cu chaperone.

Primarily energy-dependent efflux prevents cytoplasmic Cu accumulation through pumping Cu(I) across the cell membrane. As the name suggests, P-type ATPase derives cellular energy *via* hydrolyzing ATP molecules. Among five P-type ATPases, P-type I ATPase carries heavy metal as a cofactor and predominantly exists in prokaryotes. The presence of heavy metal is a significant characteristic as it determines enzymatic functionality. In the majority of prokaryotes, including SRB, P-type IB ATPase utilizes ATP for transporting the metal ions, specifically soft lewis acid, e.g., Cu^+^, Cu^2+^, Ag^+^, Zn^+^, and Pb^2+^. All P-type ATPases are membranal proteins carrying multiple domains for heavy metal binding domains and a molecular weight ranging from 70 to 150 kDa. In general, the conserved core structure of P-type enzymes consist of (i) transmembrane helix bundle (TM) necessary for substrate translocation, (ii) nucleotide-binding domain, (iii) phosphorylation domain, and (iv) actuator domain driving dephosphorylation reactions (Andersson et al., 2014). The transmembrane helix bundles, e.g., TM4, TM5, and TM6, form a high-affinity TM metal-binding site.

**Figure 4:**
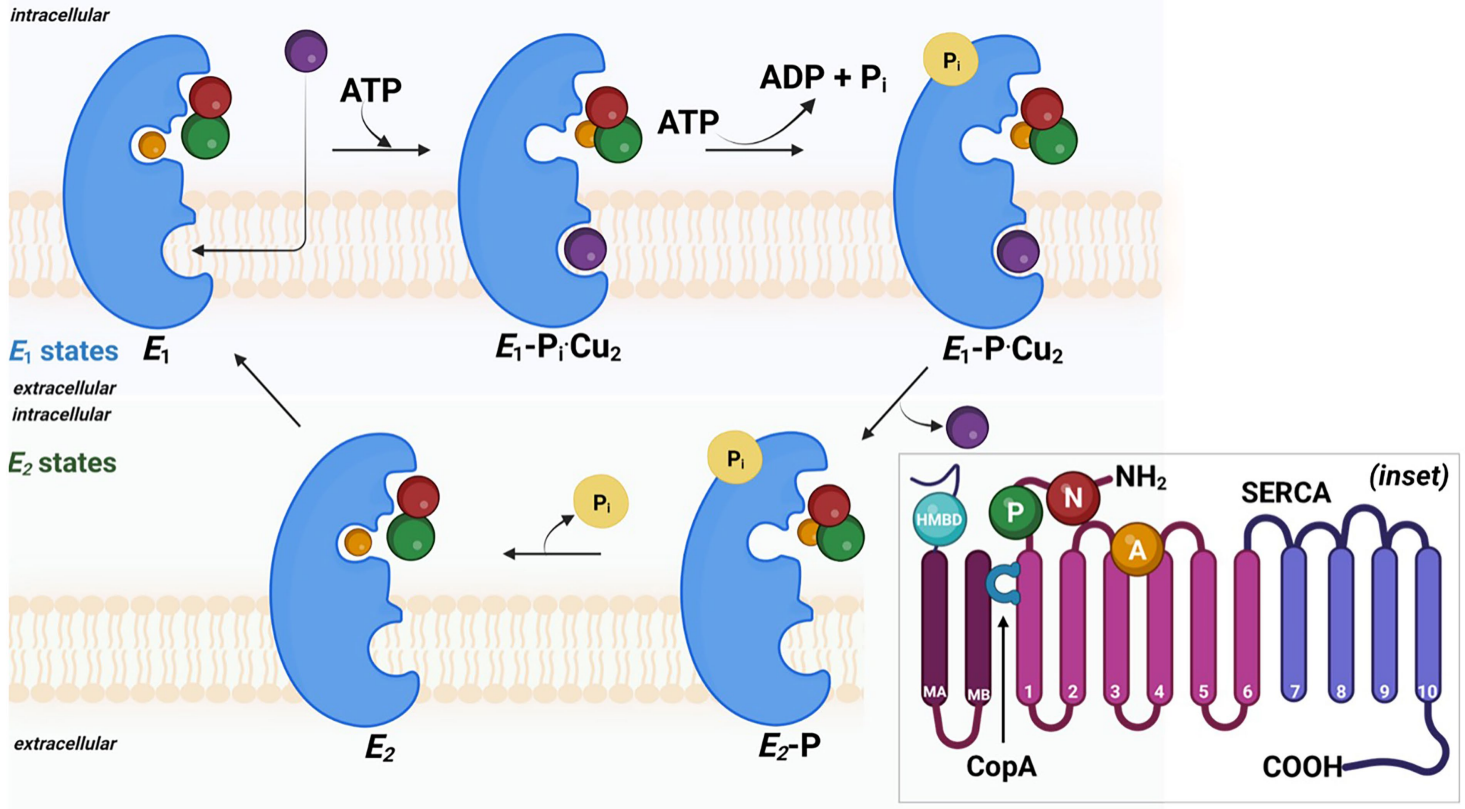
The probable mechanism involved in the copper efflux across cell membrane in sulfate reducing bacteria. Detailed 3D ribbon arrangement of different subunits and associated cofactors are shown in inset image.

The mechanistic transport of Cu(I) ions is explained by E1/E2 Albers-Post mechanism primarily emerged from studies of P-type II ATPase such as sarco(endo) plasmic reticulum Ca^2+^-ATPase (SERCA). In E1 states, intracellular ions bind with high affinity in metal-binding sites in TM from the cytoplasmic side, leading to occlusion and phosphorylation (E1P). At this stage, metals are not accessible from aqueous media E1 states, and intracellular ions bind with high affinity in metal-binding sites leading to occlusion and phosphorylation. This triggers conformational changes and access to the extracellular environment (E2P), leading to ion release and enzyme dephosphorylation (E2 state). Consequently, the E2 state shifts into inwards facing conformation and initiates a new transport cycle (Figure 4).

#### Copper sequestration

This tolerance mechanism involves intracellular sequestration of Cu(II) by high-affinity small proteins like cysteine-rich metallothioneins (CRM) and methionine sulfur residues of proteins. The CRMs are cysteine-rich metal-binding proteins and polypeptides (~7 kDa) involved in the metalloregulatory process by binding to Cu through metal thiolate clusters in their structure (Si and Lang, 2018). Their role includes intracellular distribution, storage of metals, and defense against oxidative stress. Specifically, the sulfur atom of the thiol group interacts preferentially with the heavy metals. The affinity of metal binding to metallothioneins typically follows the order of Hg^II^ > Ag^I^ > Cu^I^ > Cd^II^ > Zn^II^, all of which are part of d^10^ metal ions. Metallothioneins exhibit repeated sequence motifs of Cys-X-Cys or Cys-XX-Cys, where X stands for amino acid residues other than Cys, through which d^10^ metal ions are bound in polymetallic-thiolate cluster. A detailed mechanistic information is available on Mycobacterial metallothionein (MymT) of *Mycobacterium tuberculosis*, and counterparts in SRB are underexplored.

Similarly, S atom of methionine residue is mostly limited to Cu binding. Moreover, methionine-rich regions are common to proteins conferring Cu tolerance. For example, multicopper oxidase (CueO) of *Desulfovibrio* sp. A2 displays a methionine-rich region at T1 active site responsible for Cu binding. Fung Danny Ka et al. (2013) hypothesized that cellular amino acid (methionine) could bind and sequester intracellular Cu(I) ions. It has been observed that the addition of methionine abolished the induction of the Cus system, which is primarily responsible for Cu tolerance, and suggested that methionine plays a major role in sequestration of free and periplasmic Cu(I) (Fung Danny Ka et al., 2013). Detailed mechanistic information is available on MymT of *Mycobacterium tuberculosis*, and counterparts in SRB are underexplored.

## Copper as quorum quenchers

Quorum quenching disrupts the microbial communication channel by inducing conformational changes in the mediators and receptors. Numerous reports are available on the development of Cu stress in SRB and the consequent repression of vital genes involved in biofilm formation. The reported stress conditions result in a microcosmic scenario of limited or no microbial communications, often termed as inhibition or quenching of signaling molecules. The observed breakdown of the cellular communication network is attributed to the propensity of the heavy metals to develop metal-enzyme (e.g., CuO) nanostructure, which resulted in the deformation of the 3D signaling molecules, hence blockage of metabolic activities (Desai et al., 2021). Reports have shown small peptides and organic molecules, e.g., N-hexanoyl-L-homoserine lactone (HHL), forms a complex with Cu(II) with strong affinity. Molecular modeling of QS signaling protein of *P*. *aeruginosa* with Cu nanoparticle showed stable binding complex. LasR protein (QS protein) binded with Cu nanoparticles with a binding energy of −6.78 Kcal/mol at Glu-139 and Glu-155 (Mishra and Mishra, 2021). Organic synthesis of the QS suppressors is also reported. Gholami et al. (2020) synthesized Cu-Curcumin (Cu-Cur) complexes that halted communication mechanisms in *P*. *aeruginosa*. Compared to Cu-Cur, Fe-Cur, and Zn-Cur repressed *lasI* and *lasR* up to ~88% relative to untreated bacterial culture (Gholami et al., 2020). Chemically synthesized copper-halides, e.g., triphenylphosphane, are robust heterocyclic compounds reported to completely halt the communications channels in *P*. *aerugonisa*, *S*. *marcescens*, and *C*. *violaceum*. Despite the abundance of details on biochemical mechanisms and rate-limiting steps involved in Cu-assimilation, precise changes triggered by the Cu-ions at molecular and metabolic levels are not clearly stated.

## Quorum quenching mechanisms

**Figure 5:**
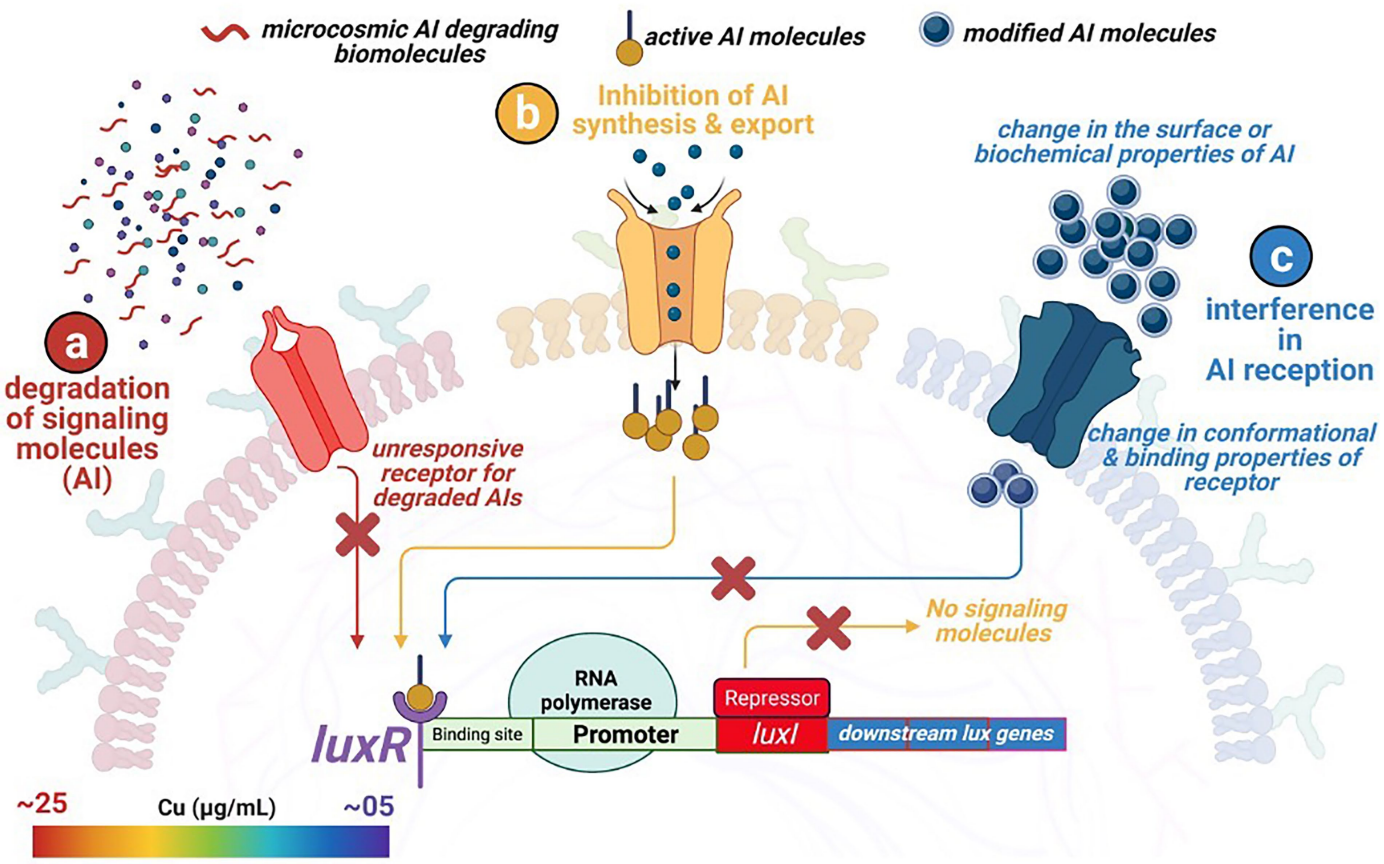
Overview of three different quorum quenching mechanisms restricting microbial attachment to external surfaces. **(a)** degradation of the signaling molecules within the microcosmic environment by competing strains and biomolecules, **(b)** no synthesis and release of the signaling molecule due to the inhibition of the regulating genes, and **(c)** change in the surface and conformational properties of the specific membranal receptors recognizing and binding with the signaling molecules.

Numerous studies decipher the QS mechanisms and their impact on microbial physiology, limited information is available on the underlying mechanisms and molecular events governing the QQ phenomenon in sulfate reducing bacteria. The roles of the Spatio-temporal factors are well defined in the microbial communication mechanisms. For example, under community competitions or nutrient scarcity conditions in SRB and *Vibrio fischeri*, LuxI protein homologs (controlling the AHL production) get upregulated, resulting in the binding of autoinducer molecules (as a ligand) with the receptor LuxR protein homologs (Figure 5). The stable interactions of the ligand-receptor molecules facilitate the synthesis and release of the N-acyl homoserine lactone (AHL) molecules, thus delineating the QS mechanisms (Utari et al., 2017). AHL concentration in the surrounding environment is sensed and bound by LuxR protein, a transcriptional regulator encoded by *luxR*, which activates the expression of *lux* operon. Transcription of *lux* gene induces more signal production in addition to the downstream expression of QS regulated genes; hence QS signal molecules are called autoinducers (AI). *Vibrio fischeri* carries the least complicated QQ machinery due to the involvement of limited mediators. A similar mechanism is expected for SRB strains. The genomic sequences of SRB report different QS regulating protein homologs, e.g., LuxS, LuxP, LuxQ, LuxO, and LuxR in *Desulfovibrio hydrothermalis*, *D*. *salexigens*, *Desulfovibrio desulfuricans*, etc. Indeed, a detailed investigation is required for precious determination of the regulators involved.

The front-line biomolecules, e.g., AHL of the QS mechanisms, are also the prime target for attaining the QQ equilibrium. Waning in the AHL biomolecules (also known as autoinducer I; AI-1) thwart synchronized gene expression and functional coordination among bacterial communities. Thus, synergistic microbial efforts such as bioluminescence, antibiotic production, pathogenicity, etc., get interposed. Therefore, an in-depth understanding of QQ mechanisms is of prime importance from an environmental, industrial, and biomedical perspective, where maintaining microbial cell density is a rate limiting step. Modified oligopeptides or autoinducer peptides (AIP) are reported to serve as AI molecules in the case of Gram-positive bacteria. In contrast, Furanosyl borate diester (*aka* Autoinducer-2) is referred to as Universal Signal Molecules due to their well-documented role in intra- and interspecies microbial communications. In the light of the reported molecular events influencing the microbial signaling pathways, the QQ scenario could be achieved through – (i) inhibiting AI synthesis and release, (ii) enzymatic degradation of AI, and (iii) interfering with AI reception by blocking receptor molecules (Figure 5).

### Inhibiting autoinducer synthesis and release

This class of QQ resulted from inhibiting the AI biosynthesis, which is relatively underreported and mediated through a complex mechanism. It is because the AHL synthesis in Gram-negative bacteria involves a complicated pathway where many factors, e.g., functional groups present in nutrients, etc., play crucial roles in microbial growth and division simultaneously. Numerous reports are highlighting the efficiency of MTA and other analogous molecules, e.g., holo-ACP, sinefungin, D/L-S-adenosylhomocysteine, L-S-adenosylcysteine, and butyryl-SAM for inhibition of AHL biosynthesis, with limited details on the underlying mechanisms. Likewise, Triclosan and 5-MAT/ S-adenosyl-homocysteine nucleosidase (MTAN) were also investigated for their impact on the inhibition of AHL biosynthesis. However, detrimental effects on amino acid and fatty acid metabolism restricted their in-depth lab investigations. Moreover, excessive dosage and prolonged exposure to these chemicals can result in the development of resistant strains of SRB.

Although the precise details on QQ mechanisms of action are unavailable. It is hypothesized that the biosynthesis of the amide bond formation between the homoserine lactone (HSL) ring and the acyl chain gets halted. *luxI* gene, which mediates the amide bond formation, also gets downregulated or disrupted. It is assumed that the eight residues of the *luxI/luxR* genes homologs in Gram-negative bacteria get mutated. Specifically, these conserved residues are involved in the intake of different acyl-ACPs, which subsequently determines the length of the acyl chain in AHL (Watson et al., 2002; Gould et al., 2004; Ortori et al., 2011). Therefore, any disruption leads to the disrupted biosynthesis of the AHL and thus results in the QQ phenomenon.

### Enzymatic degradation of autoinducers

The broad-spectrum biosynthesis of the AHL molecules by microbial communities makes it feasible to study their degradation pathways under controlled conditions. Three major subclasses of the enzymes catalyzing the AHL degradation include (i) Lactonase: which degrades AHL by opening homoserine lactone rings; (ii) Acylase: which hydrolyses the amide bond between acyl side chain and homoserine lactone rings; and (iii) Oxidoreductase: mediates oxidation or reduction of the homoserine lactones (Table 2).

**Table 2:** Classification and molecular characterization of the quorum quenching enzymes*.

Enzyme class	Enzyme name	PDB ID	Unique ligands	Amino Acids	Gene
Lactonase	Rubredoxin	1RB9	Fe_2_, SO_4_	52	*rub*, *dvu_3184*
Acylase	Ni-reconstituted hydrogenase	5JSY	C, FCO, Fe_2_, H_2_S, Ni, SF_4_	317	*hysB*, *DVU_1917*
Oxidoreductase	Ni-Fe-Se hydrogenase	6Z9G	Fe_4_ O_2_ S_4_, Cl^−^, C_3_ Fe N_2_ O, Fe_2_, H_2_S, Ni, Fe_4_ S_4_	283	*hysA*, *DVU_1918*
		2WPN	Fe_4_ O_3_ S_3,_ Fe_4_ S_4,_ C_17_ H_37_ N O_3_ S, C_3_ Fe N_2_ O, Ni^2+^, Fe^2+^, Cl^−^	317	
		3ZE9	Fe_4_ O_3_ S_3,_ Fe_4_ S_4,_ C_3_ Fe N_2_ O, Ni^2+^, Fe^2+^, Cl^−^	283	
		3ZE8	Fe_4_ S_4_, C_17_ H_38_ N O_3_ S, C_3_ Fe N_2_ O, Ni^2+^, Fe^2+^, Cl^−^	283, 484	
		3ZE7	Fe_4_ S_4,_ C_3_ Fe N_2_ O, Ni^2+^, Fe^2+^, H_2_S	283, 484	
		3ZE6	Fe_4_ O_3_ S_3_, Fe_4_ S_4,_ C_17_ H_37_ N O_3_ S, C_3_ Fe N_2_ O, C_3_ H_8_ O_3,_ Ni^2+^, Fe^2+^, Cl^−^	283, 484	
		3ZEA	Fe_4_ S_4_, C_3_ Fe N_2_ O, Ni^2+^, Fe^2+^, H_2_S	283, 484	

* Listed class of enzymes/biomolecules are identified in the *Desulfovibrio vulgaris* strain Hildenborough.

In aqueous media and at high temperatures, AHLs are prone to hydrolysis of the ester bond in lactones. Whereas in the case of acylases, 3-oxo-AHLs can transform into acylated tetramic acid by intramolecular cyclization and tetramic acid, which possess antibacterial properties. Lactone hydrolysis and tetramic acid formation show faster reaction rates with a decrease in the length of the acyl chain. In contrast, oxidoreductases generally oxidize the ω-end of the acyl chain or reduce beta-keto carbonyl of 3-oxo-AHLs. Quorum quenching of the AI-2 is a highly underexplored research area, and until recently, the only well-known enzyme to degrade AI-2 was an AI-2 kinase (LsrK) from *E*. *coli* (Pereira et al., 2013). Enzymes such as aminotransferase, 3-hydroxy-2-methylbutyry-CoA dehydrogenase, ferredoxin reductase, 4-hydroxy-3-methybut-2-en-1-yl diphosphate synthase, 3-beta hydroxysteroid dehydrogenase, or N-acetylmuramoyl-L-alanine amidase have also exhibited QQ effects against AHL and AI-2.

#### Quenching by metallo-β-lactamase like AHL lactonases

Lactoses falling under these categories are reported hydrolyzing AHL with or without substitution at C_3_’. Autoinducer inactivation enzyme (*viz*. AiiA) is the first-ever identified QQ enzyme reported to harbor zinc-binding conserved HXHXDH~60aa ~ H motif (Dong et al., 2007). The hydrolytic reaction was mediated by the two zinc ions, which polarizes the carbonyl bond of the AHL molecule, leaving it susceptible to the attack of nucleophilic water. Such an attack leads to the opening of the lactone ring by producing a carboxylic acid as the final product.

Other examples of the metallo-β-lactamase like lactonases include autoinducer inactivation enzymes (AiiA, AiiB), acyl homoserine lactonases (AhlS, AhlD, and AhlK), QS signal degradation enzyme (QsdR1), autoinducer degrading enzyme (AidC), and quorum quenching lactonase (QlcA). The majority of the reported metallo-β-lactamase like lactonases are extracted from the *Bacillus* spp., *Arthrobacter* spp., *Rhizobium* spp., and little is known about their functionalities in the SRB. The preferred substrates for these lactonases are diverse, and a few examples of substrates reported for the SRB includes N-Butanoyl L-homoserine lactone (C4-HSL), N-hexanoyl-L-Homoserine lactone (C6-HSL), N-heptanoyl-L-Homoserine lactone (C7-HSL), N-octanoyl-L-Homoserine lactone (C8-HSL), N-decanoyl-L-Homoserine lactone (C10-HSL), N-dodecanoyl-L-Homoserine lactone (C12-HSL), N-3-oxobutyryl-L-homoserine lactone (3OC4-HSL), N-3-oxohexanoyl-L-homoserine lactone (3OC6-HSL), N-3-oxo-octanoyl-L-homoserine lactone (3OC8-HSL), N-3-oxo-dodecanoyl-L-homoserine lactone (3OC12-HSL), and N-3-hydroxy-butanoyl-L-homoserine lactone (3OC4-HSL).

#### Quenching by Phosphotriesterase like AHL lactonases

Membrane cofactor protein (MCP), QS signal degradation enzyme (QsdA), and Hyper thermostable enzymes isolated from *Sulfolobus spp*. (SsoPox and SisLac) are a few reported phosphotriesterases like AHL lactonases that belong to the amidohydrolase superfamily. This class of enzyme exhibits a broad substrate specificity by acting upon C_6_- to-C_14_-HSL molecules and displays a unique degradation characteristic by preferably substituting C_3_ (the third carbon) with hydrophobic lactones. Recent reports highlighted the inherent propensity of the PLLs (Phosphotriesterase like lactonases) to serve as a QQ enzyme by improving its catalytic efficiency and altering its substrate specificity. SSPox enzyme carries a binding site for Fe^2+^ and Co^2+^ (Suzumoto et al., 2020), whereas QsdA binds to Zn^2+^. A Co^2+^ ion, after binding with SSPox interacts with the carbonyl oxygen of the lactone ring. In the case of QsdA, a catalytic mechanism similar to metallo-β-lactamase like lactonases has been reported (Uroz et al., 2008). The canonical structure of QsdA binds zinc to form a catalytic pocket that interacts with the substrate resulting in the QQ phenomenon.

#### Quenching mechanism of paraoxonases

Despite the mammalian origin of the paraoxonases (PONs), their broad substrate specificity and occurrence in a diverse array of microbial genera have been reported. PONs can hydrolyze C7-, C12-, N-tetradecanoyl-L-Homoserine lactone C14-HSL, 3OC6-, 3OC10-, and 3OC12-HSL. It is reported that PON1 and PON3 are present in the serum associated with high-density lipoprotein, whereas PON2 works as intracellular enzymes. The primary role of the PONs is to provide the first line of defense to the mammalian host against microbial invaders relying upon the AHL-mediated communication mechanisms. The study of the crystal structure of PON1 has revealed that it can bind with two Ca^2+^ ions in its tunnel-shaped binding pocket of the central cavity. One of the Ca^+^ ions plays a significant role in the lactone ring degradation mechanism by interacting with the water molecule and phosphate (Harel et al., 2004). Although paraoxonases have been proven to work as QQ, their presence in sulfate reducing bacteria is still not reported and should be explored in future endeavors.

#### Quenching mechanism of AHL acylases

The AHL acylase belongs to the N-terminal nucleophile (Ntn) hydrolase superfamily and prefers the long-chain AHL molecules. AHL degrading enzyme (AhlM), autoinducer binding protein (AibP), autoinducer inhibiting enzyme (AiiD, AiiC, and AiiO), and Pyoverdine destructing enzyme (PvdQ) are a few AHL acylases reported in *Streptomyces*, *Brucella*, *Ralstonia*, *Shewanella*, *Ochrobactrum*, and a few SRB species. Amylases break the amide bond present in AHL and form HSL (Homoserine lactone), which in turn serves as a source of N- and fatty acid to support microbial energy needs (Park et al., 2005). The role of the AHL acylases predominates over lactonases in the QQ mechanisms. It is due to the fact that acylases mediated degradation products are incompatible with self-assembly; thus, no spontaneous generation of the signaling molecules occurs (Fetzner, 2015). Studies have found that PvdQ has an αββα-fold structure, and autoproteolysis creates an N-terminal serine which serves as a nucleophile for its catalytic event after being activated by a water molecule. The serine hydroxide loses its proton due to the hydrogen bonding between terminal α-amine and the water molecule. Activated hydroxylate forms a reactive oxyanion with the carbonyl carbon of the amide bond of AHL, and proton from the water molecule binds with the nitrogen of the amide, which releases HSL from the AHL molecule. AlhM and AiiD also show similar catalytic activity like PvdQ with preference over different acyl chain lengths.

#### Quenching mechanism of AHL oxidoreductase

Oxidoreductases generally oxidize the ω-end of the acyl chain or reduce beta-keto carbonyl of 3-oxo-AHLs. The cytochrome P_450_BM-3 can hydroxylate AHLs, the corresponding N-acyl homoserines can act as less active signaling molecules. Besides, oxidoreductase BpiB09 and aldehyde dehydrogenase AldR can also modify AHLs and reduces motility and biofilm formation. BpiB01, BpiB04, and BpiB07 have been observed to degrade 3-oxo-C_8_-HSL with a low concentration of zinc or calcium. Until now, 710 AHL lactonases have been identified, and only one lactonase, which is Rubredoxin, has been recognized for *Desulfovibrio Vulgaris* Hildenborough, which has ligand specificity toward Fe^2+^ and SO_4_^−^. According to PDB, eight acylase and oxidoreductases have been identified for *Desulfovibrio* spp. sulfate reducing bacteria like *Desulphovibrio* spp. has been noted to accelerate microbially induced corrosion significantly (Tables 2, 3). These enzymes have proved to reduce corrosion up to ~50% with improved efficiency compared to chemical and biochemical compounds (Huang et al., 2019).

**Table 3:** Overview of significant characteristics reported for different strains of the sulfate reducing bacteria.

Species	Growth medium	Copper toxicity	MIC^§^	QS^⊥^ homologs	QQ^‡^ homolog	Nanowires
*Desulfovibrio desulfuricans*	Basal bicarbonate media lactate/nitrate media	~49% growth reduction at 20 ppm	Stainless steel	Two component transcriptional regulator protein, LuxR family (SAMN02910291_00253) LuxR regulators (DDIC_04350, DDE01_10870)	MBL* hydrolase (DDE01_19620)	Reported; gene IDs not annotated
*Desulfovibrio vulgaris* str. Hildenborough	Lactate C media Widdel-pfennig media	Complete inhibition at ~5 mM	Iron ~20 milli-inch per year (mpy)	LuxR (DVU_2675, DVU_2577)	MBL (DVU_2310)	Reported; DVU0797, DVU0799
*Oleidesulfovibrio alaskensis* G20	Lactate C media lactate sulfate (LS4D) media	15 μM resulted in 1.7-fold decrease in growth rate	Copper up to 4 mpy	AI 2 binding periplasmic protein LuxP precursor (Dde_3311) LuxR family (Dde_2674, Dde_0977)	NR	NR
*Desulfovibrio ferrophilus* IS5	DSMZ195c media ASW media	NR	Carbon steel ~0.27 (mpy)	AI 2 binding periplasmic protein LuxP (DFE_2651) two components transcriptional regulator LuxR family (DFE_3273, DFE_2913)	NR	Reported; DFE_0465
*Desulfovibrio gigas*	Lactate-Sulfate medium Formate-acetate-sulfate	Complete inhibition at ~1 mM	Mild steel	NA	NR	NR
*Desulfosporosinus orientis*	DSMZ # 63	NR	Mild Steel	LuxR C-terminal-related transcriptional regulator (DESOR_RS26240)	NR	Reported
*Desulfovibrio Caledoniensis*	Postage-C media	NR	Reported High strength steel	NR	NR	Reported
*Desulfovibrio vulgaris* RCH1	Lactate sulfate media	NR	NR	Two-component transcriptional regulator, LuxR family (Deval_2379, Deval_2467, Deval_2487)	MBL (Deval_2141)	Reported; SEM images
*Desulfovibrio* sp. A2	Basal salt media	Tolerate ~40 mM without affecting growth rate	NR	LuxR (DA2_1048, DA2_1152)	MBL (DA2_3744)	NR

§ Microbially induced corrosion reported for.

⊥ Quorum sensing.

‡ Quorum quenching.

* Metallo-beta-lactamase protein; NR: not reported.

### Interfering autoinducer recognition and binding

Compounds with similar structures as autoinducers, such as halogenated furanone, vanillin, malic, and lactic acid, have been reported to interfere with the signal receptor. Chemical synthesis studies targeting the structural alteration of the signaling molecules are focused on either the acyl side chain or the lactone ring. In a few reported instances, e.g., Blackwell et al. (2010) have designed a few AHL analogs which compete against native AHLs and synthetic molecules which can receive QS molecules instead of actual receptors and disrupt the overall QS phenomenon. Iyer et al. (2013) and Amara et al. (2009) have also manufactured a few QS signal analogs with limited success in their role as QQ (Amara et al., 2009; Iyer et al., 2013). Persson et al. (2005) have shown that LuxR expression can be blocked by substituting C-3 atom with Sulphur in an acyl chain (Persson et al., 2005). By replacing the aromatic structure of the AHL molecules with an aryl structure of almost similar size, quorum quenching can be activated, which by replacing C-1 carbonyl of the side chain with sulphonyl group can be further supplemented. In contrast, structural modifications such as additional atoms in the lactone ring and additional moieties in C-3 atom resulted in the nonfunctional AHL analogs working as a QQ.

## Machine learning tools for anti-biofilm peptides

As the name suggests, anti-biofilm peptides are natural or synthetic peptides that disrupt the autoinducers and halt biofilm formation. Several such peptides have been identified in all forms of life and can be found in the antimicrobial peptide database. In contrast, antibiofilm peptides are a subset of the antimicrobial peptide database and are listed in the antibiofilm peptide database. However, extracting and evaluating a peptide to be classified as these peptides experimentally has been a major challenge. The ML has been used to establish a general trend and pattern from existing biofilm peptides that enables the classification of peptides for their potential inhibitory properties (Gupta et al., 2016; Srivastava et al., 2020). ML has been an efficient and robust statistical tool to make a prediction in various contexts, including protein function prediction, antibiofilm agents, QS molecules, and many more, using the existing data. ML can be divided into supervised and unsupervised methods (Sarker, 2021). Supervised ML relies on existing data and measured outcomes to build a prediction model. A number of inputs or features characterizes each input, and the presence of an outcome guides the ML process (Sarker, 2021). In comparison, the unsupervised learning approach does not rely on the outcome and depends purely on input features to predict. In general, an algorithm is trained with a large relevant dataset to learn and correlate the features of the input dataset and prediction task. For this purpose, numerous algorithms such as Random Forest (RF), Support-vector machine (SVM), and Neural Networks. Moreover, using ML or computational methods to identify or predict would reduce the experimental identification procedure, which is generally time-consuming, challenging, and tedious. Not limited to the prediction of QQ molecules, ML has been used to construct the QS communication network for human gut microbiota (Wu et al., 2022), predict QS peptides, to recognize phenotype, and many more.

Several studies have reported successfully predicting the antibiofilm peptides using ML models. Similarly, peptide sequences were experimentally validated to extract the sequence-based features to construct a support vector machine-based prediction tool with an accuracy of 97.83% (Gupta et al., 2016). Similarly, ~122 molecules known for antimicrobial properties from either plant, human, or synthetic as a positive dataset and included 189 metabolites involved in biochemical pathways as a negative dataset to extract structural and chemical features and train the number of ML algorithms such as RF, SVM to predict an independent set of data for its biofilm inhibitory properties (Srivastava et al., 2020). ML has not only been limited to predicting the anti-biofilm peptides but they are also used to design anti-biofilm peptides.

**Figure 6:**
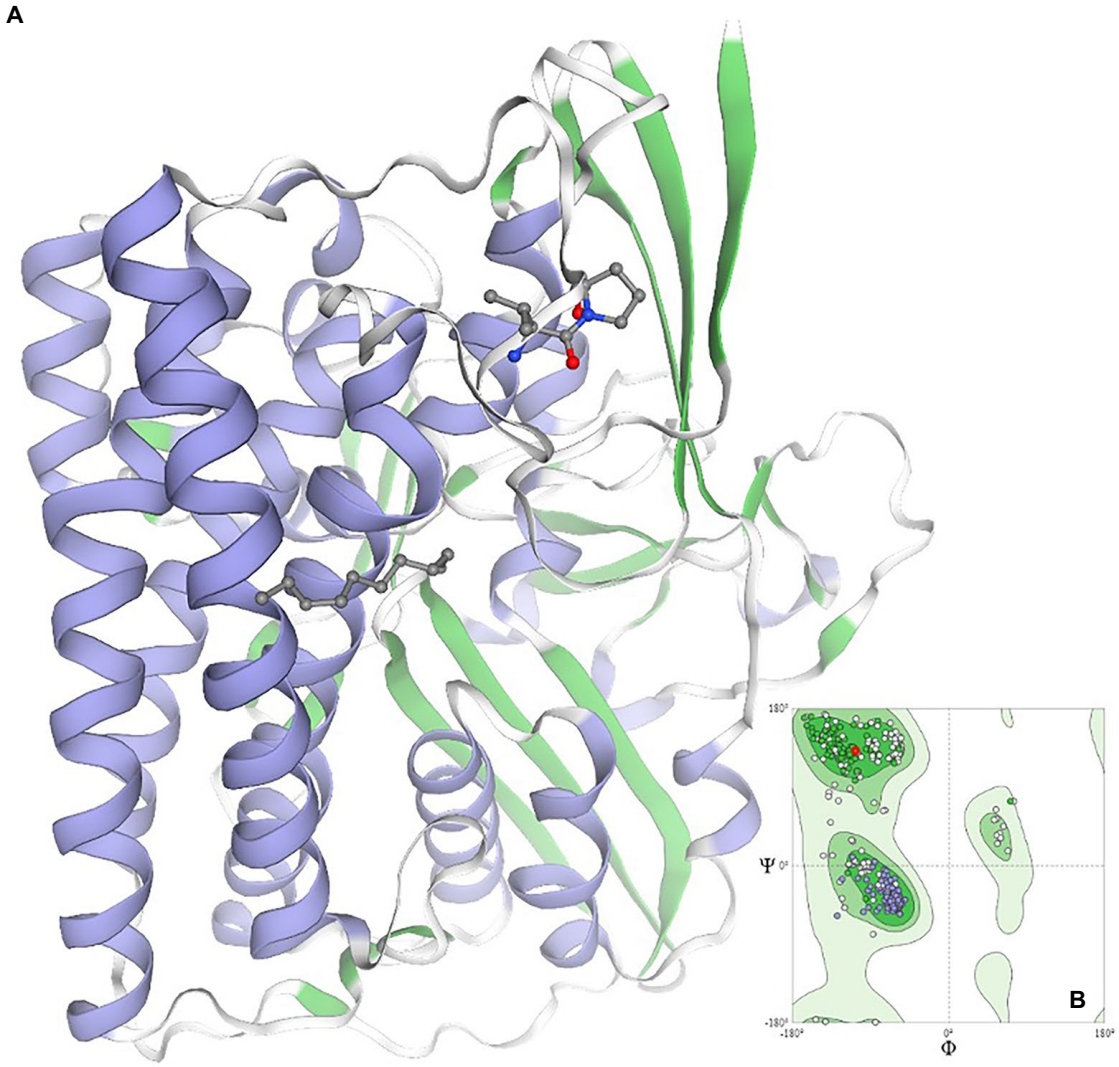
Homology modeling of the quorum quenching homolog in the case of *Oleidesulfovibrio alaskensis* G20*. Ramachandran plot is given as an inset image validating the structural conformation of the model as >98% of residues exist in the favored region *quorum quenching molecule encoded by gene ID Dde_2135 in *Oleidesulfovibrio alaskensis* G20 is homolog of HysB of *Desulfovibrio vulgaris*.

Using data mining and data curing tools, the networking and interactions of the significant gene *viz*. Dde_3311, serving as the autoinducer (*LuxP*), was identified. The identified gene is critical for designing the new anti-biofilm peptide due to its annotated role and its significant interactions with at least two genes, e.g., Dde_0544 and Dde_0849, which are directly involved in cellular communications (Supplementary Figure 1). The gene Dde_849 is annotated to encode for exosortase type proteins, which are reported to harbor the Pro-Glu-Pro domain for their positioning as the trans-membranal proteins. The roles of transmembranal proteins are critical to maintaining cellular communications by controlling their permeability properties. Additionally, the close regulation of the gene, e.g., Dde_1055 which is an ATP binding cassette gene, highlights the regulation of energy transfer and energy balance associated with the signaling mechanisms of the microbe. It is evident that colonization and spreading of the microbial biofilm require higher cellular energy levels; thus, the upregulation of the Dde_1055 is expected. For anti-biofilm peptides, next-generation sequencing techniques could be adopted to design the truncated *LuxP* gene and to study its expression. Likewise, using the computational biology tools, pioneering information pertaining to the QQ homolog in *D*. *alaskensis* G20 has been predicted and modeled (Figure 6). The validity of the modeled structure is validated using the Ramachandran plot, and ~ 99% of the residues are in the permissible area, thus validating the structure of the QQ homolog.

Most ML models are supervised, requiring either a positive dataset (anti-biofilm) or a negative dataset (not anti-biofilm). The prediction output would generally classify an independent/test dataset as anti-biofilm or non-anti-biofilm. Hence, ML algorithms are sometimes referred to as classifiers as well. ML depends upon the extracted features from the sequence of proteins or peptides. Some sequence-based features have been widely used as composition features (amino acid composition, and dipeptide composition), amino acid distance-based features (composition, transition, distribution), and physiochemical properties. Tools *viz*. SPiCE, iFeatures, MathFeatures, Seq2Feature, and IAMPE are web-based tools to aid in the extraction of features. Hence, it is essential to select an appropriate feature to improve the prediction model’s accuracy.

## Potential development and future directions

As highlighted in the earlier sections of the review, many physiological facets of the SRB and biofilm development are underexplored. Considering reviewed literature, the three key aspects which will be transformative for future research on microbial attachment and communications are (i) characterization of the conditioning film components, (ii) Cu-sequestration mechanisms, and (iii) applications of computational biology and ML.

The authoritative impact of the constituent biomolecules of conditioning film on planktonic cells needs more attention. Specifically, constituents other than glycolipids and carbohydrates require detailed investigation for their structural and functional properties. Considering the structural diversity and functional mediation offered by a protein molecule, their significant impact is projected on the guided chemotaxis movement of the planktonic cells towards external surfaces. Therefore, future research could be dominated by the whole proteome analysis and time-dependent transcriptome analysis. In addition, the role of analytical techniques, e.g., ion-chromatography, and LC/MS for metabolic flux analysis, is equally important to correlate the microbial growth and stress mechanisms with the underexplored phenotypes, e.g., microbial nanowire developments.

Detailed mechanistic understanding of a few enzyme families, *viz*. multicopper oxidases, P-type IB ATPase, etc., is quintessential to comprehending the Cu-sequestration mechanisms. Understanding the active mechanism involved in converting Cu(I) to less toxic Cu(II) is critical in understanding on microbial physiology under stress conditions. It is also important to decode the roles of enzymes involved in the development of the Cu nanostructures, which disrupts the microbial communication mechanisms. Early reports indicate the plausible roles of the P-type IB ATPase enzymes in Cu-tolerances.

The scope of computational biology, ML, and artificial intelligence will be unparalleled in decoding biological phenomena, including microbial “Rules of Life.” The ML tools, e.g., SVM, hidden Markov models (HMM), etc., will be critical in predicting the structural details. Likewise, bidirectional recursive neural networks (BRNN), RF algorithms, etc., will be the key to depicting the biomolecules (proteins, lipids), ligands, and metal–ligand’s complexes. The ML-guided biomanufacturing of the novel quenchers will leads to the synthesis of robust compounds to tackle the major environmental and industrial challenges such as MIC, membrane biofouling, etc. The ML/artificial intelligence, molecular dynamics, and quantum mechanics tools will open a new horizon for studying substrate docking and binding specificities.

## Conclusion

Identification of ~700 microbial autoinducers corroborates a clear understanding of the later stages of microbial colonization resulting in a mature biofilm. In contrast, no structural and functional description is available on conditioning proteins regulating the first irreversible attachments of planktonic cells in natural landscapes. This extreme disparity in knowledge highlights the distinctive gap in scientific understanding of SRB’s communication channels and coping mechanisms active during stress conditions. The classification of the conditioning film’s component is the critical step in identifying the molecular determinants of microbial attachment. Precise monitoring of the microbial profile and microcosmic conditions is the prerequisite, which is otherwise rate-limiting variables in accurately characterizing the biomolecules. Likewise, given the diverse functionality of the Cu-ions in SRB growth and communication channels, an updated understanding of its sequestration pathways is needed. With the advent of the ML tools, numerous robust algorithms are under testing to annotate unexplored assignments of the Cu-ions. In addition, the central role of Cu as a cofactor in metabolic enzymes necessitates monitoring metabolic flux to establish a correlation between nutritional stress and biofilm formation by SRB. Revisiting the metabolic phenotypes will assist in biomanufacturing the Green solutions to prevent the catastrophic impacts of MIC caused by the SRB biofilms.

## Author contributions

DR and AS: conceptualization and writing. AK, SG, DS, EG, and VG: conceptualization. SD: conceptualization, writing, and editing. All authors contributed to the article and approved the submitted version.

## Funding

The authors acknowledge the financial support provided by the National Science Foundation (NSF) Award # 1849206 as 2DBEST Research Center and the Governor Office of Economic Development as CNAM-BIO Research Center. SD acknowledges the financial support provided by the Nelson Research fund and SD EPSCOR as a Competitive Research Grant for a preliminary understanding of the conditioning proteins. DR acknowledges the partial financial support provided by the NSF Award # 1920954 as DDMD project.

## Conflict of interest

The authors declare that the research was conducted in the absence of any commercial or financial relationships that could be construed as a potential conflict of interest.

## Publisher’s note

All claims expressed in this article are solely those of the authors and do not necessarily represent those of their affiliated organizations, or those of the publisher, the editors and the reviewers. Any product that may be evaluated in this article, or claim that may be made by its manufacturer, is not guaranteed or endorsed by the publisher.

## Supplementary material

Supplementary Figure 1 Overview of molecular interactions and networking among all essential genes involved in the autoinducer release in *Oleidesulfovibrio alaskensis* G20.

## References

[ref1] Amara N. Mashiach R. Amar D. Krief P. Spieser S. A. Bottomley M. J.. (2009). Covalent inhibition of bacterial quorum sensing. J. Am. Chem. Soc. 131, 10610–10619. doi: 10.1021/ja903292v, PMID: 19585989

[ref2] Andersson M. Mattle D. Sitsel O. Klymchuk T. Nielsen A. M. Møller L. B.. (2014). Copper-transporting P-type ATPases use a unique ion-release pathway. Nat. Struct. Mol. Biol. 21, 43–48. doi: 10.1038/nsmb.2721, PMID: 24317491 PMC3904665

[ref3] Arnaouteli S. MacPhee C. E. Stanley-Wall N. R. (2016). Just in case it rains: building a hydrophobic biofilm the Bacillus subtilis way. Curr. Opin. Microbiol. 34, 7–12. doi: 10.1016/j.mib.2016.07.012, PMID: 27458867

[ref4] Baker J. Sitthisak S. Sengupta M. Johnson M. Jayaswal R. K. Morrissey J. A. (2010). Copper stress induces a global stress response in <i>Staphylococcus aureus</i> and represses *sae* and *agr* expression and biofilm formation. Appl. Environ. Microbiol. 76, 150–160. doi: 10.1128/AEM.02268-09, PMID: 19880638 PMC2798663

[ref5] Bakker D. P. Busscher H. J. van Zanten J. de Vries J. Klijnstra J. W. van der Mei H. C. (2004). Multiple linear regression analysis of bacterial deposition to polyurethane coatings after conditioning film formation in the marine environment. Microbiology 150, 1779–1784. doi: 10.1099/mic.0.26983-0, PMID: 15184564

[ref6] Bakker D. P. Klijnstra J. W. Busscher H. J. van der Mei H. C. (2003). The effect of dissolved organic carbon on bacterial adhesion to conditioning films adsorbed on glass from natural seawater collected during different seasons. Biofouling 19, 391–397. doi: 10.1080/08927010310001634898, PMID: 14768468

[ref7] Berne C. Ellison C. K. Ducret A. Brun Y. V. (2018). Bacterial adhesion at the single-cell level. Nat. Rev. Microbiol. 16, 616–627. doi: 10.1038/s41579-018-0057-5, PMID: 30008468

[ref8] Bhagwat G. O'Connor W. Grainge I. Palanisami T. (2021). Understanding the fundamental basis for biofilm formation on plastic surfaces: role of conditioning films. Front. Microbiol. 12:687118. doi: 10.3389/fmicb.2021.687118, PMID: 34248907 PMC8267902

[ref9] Bhosle N. B. Garg A. Fernandes L. Citon P. (2005). Dynamics of amino acids in the conditioning film developed on glass panels immersed in the surface seawaters of Dona Paula Bay. Biofouling 21, 99–107. doi: 10.1080/08927010500097821, PMID: 16167390

[ref001] Blackwell H. E. Geske G. D. Wezeman R. W. (2010). Compounds and methods for modulating communication and virulence in quorum sensing bacteria. Google Patents.

[ref10] Cabrera G. Pérez R. Gómez J. M. Ábalos A. Cantero D. (2006). Toxic effects of dissolved heavy metals on Desulfovibrio vulgaris and Desulfovibrio sp. strains. J. Hazard. Mater. 135, 40–46. doi: 10.1016/j.jhazmat.2005.11.058, PMID: 16386832

[ref11] Chatterjee M. Fan Y. Cao F. Jones A. A. Pilloni G. Zhang X. (2021). Proteomic study of Desulfovibrio ferrophilus IS5 reveals overexpressed extracellular multi-heme cytochrome associated with severe microbiologically influenced corrosion. Sci. Rep. 11:15458. doi: 10.1038/s41598-021-95060-0, PMID: 34326431 PMC8322314

[ref12] Chen Z. Gao S.-h. Jin M. Sun S. Lu J. Yang P.. (2019). Physiological and transcriptomic analyses reveal CuO nanoparticle inhibition of anabolic and catabolic activities of sulfate-reducing bacterium. Environ. Int. 125, 65–74. doi: 10.1016/j.envint.2019.01.058, PMID: 30710801

[ref13] Chilkoor G. Jawaharraj K. Vemuri B. Kutana A. Tripathi M. Kota D.. (2020). Hexagonal boron nitride for sulfur corrosion inhibition. ACS Nano 14, 14809–14819. doi: 10.1021/acsnano.0c03625, PMID: 33104334

[ref14] Compère C. Bellon-Fontaine M. N. Bertrand P. Costa D. Marcus P. Poleunis C.. (2001). Kinetics of conditioning layer formation on stainless steel immersed in seawater. Biofouling 17, 129–145. doi: 10.1080/08927010109378472

[ref15] Copper S. M. (2018). Toxicity. Copper and Bacteria. Cham: Springer International Publishing, 11–19.

[ref16] Desai D. G. Swarali H. Navale G. R. Prabhune A. Late D. J. Dharne M. S.. (2021). Inhibition of quorum sensing, motility and biofilm formation of Pseudomonas aeruginosa by Copper oxide nanostructures. J. Clust. Sci. 32, 1531–1541. doi: 10.1007/s10876-020-01916-2

[ref17] Dong Y.-H. Wang L.-H. Zhang L.-H. (2007). Quorum-quenching microbial infections: mechanisms and implications. Philos. Trans. Roy. Soc. B. Biol. Sci. 362, 1201–1211. doi: 10.1098/rstb.2007.2045, PMID: 17360274 PMC2435583

[ref18] Fetzner S. (2015). Quorum quenching enzymes. J. Biotechnol. 201, 2–14. doi: 10.1016/j.jbiotec.2014.09.001 25220028

[ref19] Fichtel K. Mathes F. Könneke M. Cypionka H. Engelen B. (2012). Isolation of sulfate-reducing bacteria from sediments above the deep-subseafloor aquifer. Front. Microbiol. 3:65. doi: 10.3389/fmicb.2012.00065, PMID: 22363336 PMC3282481

[ref20] Fong J. N. C. Yildiz F. H. (2015). Biofilm matrix proteins. Microbiol. Spectr. 3:2. doi: 10.1128/microbiolspec.MB-0004-2014, PMID: 26104709 PMC4480581

[ref21] Foster T. J. (2020). Surface proteins of Staphylococcus epidermidis. Front. Microbiol. 11:1829. doi: 10.3389/fmicb.2020.01829, PMID: 32849430 PMC7403478

[ref22] Francius G. El Zein R. Mathieu L. Gosselin F. Maul A. Block J.-C. (2017). Nano-exploration of organic conditioning film formed on polymeric surfaces exposed to drinking water. Water Res. 109, 155–163. doi: 10.1016/j.watres.2016.11.038, PMID: 27883920

[ref23] Friedlander R. S. Vlamakis H. Kim P. Khan M. Kolter R. Aizenberg J. (2013). Bacterial flagella explore microscale hummocks and hollows to increase adhesion. Proc. Natl. Acad. Sci. U. S. A. 110, 5624–5629. doi: 10.1073/pnas.1219662110, PMID: 23509269 PMC3619351

[ref24] Fung Danny Ka C. Lau Wai Y. Chan Wing T. Yan A. (2013). Copper efflux is induced during anaerobic amino acid limitation in Escherichia coli to protect iron-sulfur cluster enzymes and biogenesis. J. Bacteriol. 195, 4556–4568. doi: 10.1128/JB.00543-13, PMID: 23893112 PMC3807430

[ref25] Garg A. Jain A. Bhosle N. B. (2009). Chemical characterization of a marine conditioning film. Int. Biodeterior. Biodegradation 63, 7–11. doi: 10.1016/j.ibiod.2008.05.004

[ref26] Gholami M. Zeighami H. Bikas R. Heidari A. Rafiee F. Haghi F. (2020). Inhibitory activity of metal-curcumin complexes on quorum sensing related virulence factors of Pseudomonas aeruginosa PAO1. AMB Express 10:111. doi: 10.1186/s13568-020-01045-z, PMID: 32514786 PMC7280416

[ref27] Gilbert P. Collier P. J. Brown M. (1990). Influence of growth rate on susceptibility to antimicrobial agents: biofilms, cell cycle, dormancy, and stringent response. Antimicrob. Agents Chemother. 34, 1865–1868. doi: 10.1128/AAC.34.10.1865, PMID: 2291653 PMC171955

[ref28] Gorbushina A. A. (2007). Life on the rocks. Environ. Microbiol. 9, 1613–1631. doi: 10.1111/j.1462-2920.2007.01301.x 17564597

[ref29] Gould T. A. Schweizer H. P. Churchill M. E. (2004). Structure of the Pseudomonas aeruginosa acyl-homoserinelactone synthase LasI. Mol. Microbiol. 53, 1135–1146. doi: 10.1111/j.1365-2958.2004.04211.x, PMID: 15306017

[ref30] Gramp J. P. Sasaki K. Bigham J. M. Karnachuk O. V. Tuovinen O. H. (2006). Formation of Covellite (CuS) under biological sulfate-reducing conditions. Geomicrobiol J. 23, 613–619. doi: 10.1080/01490450600964383

[ref31] Grubman A. White A. R. (2014). Copper as a key regulator of cell signalling pathways. Expert Rev. Mol. Med. 16:e11. doi: 10.1017/erm.2014.11, PMID: 24849048

[ref32] Gupta S. Sharma A. K. Jaiswal S. K. Sharma V. K. (2016). Prediction of biofilm inhibiting peptides: an in silico approach. Front. Microbiol. 7:949. doi: 10.3389/fmicb.2016.00949, PMID: 27379078 PMC4909740

[ref33] Hagiwara T. Sakiyama T. Fau W. H. (2009). Molecular simulation of bovine beta-lactoglobulin adsorbed onto a positively charged solid surface. (0743–7463 (Print)). 10.1021/la8024149 19032076

[ref34] Hao O. J. Huang L. Chen J. M. Buglass R. L. (1994). Effects of metal additions on sulfate reduction activity in wastewaters. Toxicol. Environ. Chem. 46, 197–212. doi: 10.1080/02772249409358113

[ref35] Harel M. Aharoni A. Gaidukov L. Brumshtein B. Khersonsky O. Meged R.. (2004). Structure and evolution of the serum paraoxonase family of detoxifying and anti-atherosclerotic enzymes. Nat. Struct. Mol. Biol. 11, 412–419. doi: 10.1038/nsmb767, PMID: 15098021

[ref36] Harjai K. Sabharwal N. (2017). Biofilm formation and quorum sensing in rhizosphere. Biofilms Plant Soil Health, 111–130. doi: 10.1002/9781119246329.ch7

[ref37] Huang S. Bergonzi C. Schwab M. Elias M. Hicks R. E. (2019). Evaluation of biological and enzymatic quorum quencher coating additives to reduce biocorrosion of steel. PLoS One 14:e0217059. doi: 10.1371/journal.pone.0217059, PMID: 31095643 PMC6522020

[ref38] Hussain A. Hasan A. Javid A. Qazi J. I. (2016). Exploited application of sulfate-reducing bacteria for concomitant treatment of metallic and non-metallic wastes: a mini review. 3 Biotech 6:119. doi: 10.1007/s13205-016-0437-3 PMC4902799 28330194

[ref002] Hwang G. Liang J. Kang S. Tong M. Liu Y. (2013). The role of conditioning film formation in Pseudomonas aeruginosa PAO1 adhesion to inert surfaces in aquatic environments. Biochem. Eng. J. 76, 90–98.

[ref39] Hyre A. Casanova-Hampton K. Subashchandrabose S. (2021). Copper homeostatic mechanisms and their role in the virulence of Escherichia coli and salmonella enterica. EcoSal Plus 9:eESP00142020. doi: 10.1128/ecosalplus.ESP-0014-2020, PMID: 34125582 PMC8669021

[ref40] Iyer R. S. Ganguly K. Silks L. A. (2013). Synthetic analogs of bacterial quorum sensors. Google Patents.

[ref41] Jain A. Bhosle N. B. (2009). Biochemical composition of the marine conditioning film: implications for bacterial adhesion. Biofouling 25, 13–19. doi: 10.1080/08927010802411969, PMID: 18846459

[ref42] Jia R. Yang D. Xu D. Gu T. (2018). Carbon steel biocorrosion at 80 °C by a thermophilic sulfate reducing archaeon biofilm provides evidence for its utilization of elemental iron as electron donor through extracellular electron transfer. Corros. Sci. 145, 47–54. doi: 10.1016/j.corsci.2018.09.015

[ref43] Kalin M. Wheeler W. N. Bellenberg S. (2018). Acid rock drainage or not—oxidative vs reductive biofilms—a microbial question. Fortschr. Mineral. 8:199. doi: 10.3390/min8050199

[ref44] Kaur K. Sharma A. Capalash N. Sharma P. (2019). Multicopper oxidases: biocatalysts in microbial pathogenesis and stress management. Microbiol. Res. 222, 1–13. doi: 10.1016/j.micres.2019.02.007, PMID: 30928025

[ref45] Khatoon Z. McTiernan C. D. Suuronen E. J. Mah T.-F. Alarcon E. I. (2018). Bacterial biofilm formation on implantable devices and approaches to its treatment and prevention. Heliyon 4:e01067. doi: 10.1016/j.heliyon.2018.e01067, PMID: 30619958 PMC6312881

[ref46] Kostakioti M. Hadjifrangiskou M. Hultgren S. J. (2013). Bacterial biofilms: development, dispersal, and therapeutic strategies in the dawn of the postantibiotic era. Cold Spring Harb. Perspect. Med. 3:a010306-a. doi: 10.1101/cshperspect.a010306 23545571 PMC3683961

[ref47] Kukulka D. J. Baier R. Mollendorf J. (2004). Factors associated with fouling in the process industry. Heat Transfer Eng. 25, 23–29. doi: 10.1080/01457630490458978

[ref48] Ladomersky E. Petris M. J. (2015). Copper tolerance and virulence in bacteria. Metallomics 7, 957–964. doi: 10.1039/C4MT00327F, PMID: 25652326 PMC4464932

[ref49] Loeb G. I. Neihof R. A. (1975). Marine Conditioning Films. Applied Chemistry at Protein Interfaces. Advances in Chemistry, vol. 145 American Chemical Society, 319–335.

[ref50] Lorite G. S. Rodrigues C. M. de Souza A. A. Kranz C. Mizaikoff B. Cotta M. A. (2011). The role of conditioning film formation and surface chemical changes on Xylella fastidiosa adhesion and biofilm evolution. J. Colloid Interface Sci. 359, 289–295. doi: 10.1016/j.jcis.2011.03.066, PMID: 21486669

[ref51] Mancini S. Kumar R. Mishra V. Solioz M. (2017). Desulfovibrio DA2_CueO is a novel multicopper oxidase with cuprous, ferrous and phenol oxidase activity. Microbiology 163, 1229–1236. doi: 10.1099/mic.0.000509, PMID: 28749328

[ref52] Mayilraj S. Kaksonen A. H. Cord-Ruwisch R. Schumann P. Spröer C. Tindall B. J.. (2009). Desulfonauticus autotrophicus sp. nov., a novel thermophilic sulfate-reducing bacterium isolated from oil-production water and emended description of the genus Desulfonauticus. Extremophiles 13, 247–255. doi: 10.1007/s00792-008-0212-4, PMID: 19050820

[ref003] Miao L. Wang C. Adyel T. M. Zhao J. Yan N. Wu J.. (2021). Periphytic biofilm formation on natural and artificial substrates: Comparison of microbial compositions, interactions and functions. Front. Microbiol. 12:1917. 10.3389/fmicb.2021.684903 PMC8350161 34381427

[ref53] Mishra A. Mishra N. (2021). Antiquorum sensing activity of Copper nanoparticle in pseudomonas aeruginosa: an in silico approach. Proc. Nat. Acad. Sci. India Sec. B. Biol. Sci. 91, 29–36. doi: 10.1007/s40011-020-01193-z

[ref54] Muhammad M. H. Idris A. L. Fan X. Guo Y. Yu Y. Jin X.. (2020). Beyond risk: bacterial biofilms and their regulating approaches. Front. Microbiol. 11:928. doi: 10.3389/fmicb.2020.00928, PMID: 32508772 PMC7253578

[ref55] Nadell C. D. Xavier J. B. Levin S. A. Foster K. R. (2008). The evolution of quorum sensing in bacterial biofilms. PLoS Biol. 6:e14. doi: 10.1371/journal.pbio.0060014, PMID: 18232735 PMC2214811

[ref56] Nan L. Xu D. Gu T. Song X. Yang K. (2015). Microbiological influenced corrosion resistance characteristics of a 304L-cu stainless steel against Escherichia coli. Mater. Sci. Eng. C Mater. Biol. Appl. 48, 228–234. doi: 10.1016/j.msec.2014.12.004, PMID: 25579918

[ref57] Nandakumar V. Chittaranjan S. Kurian V. M. Doble M. (2013). Characteristics of bacterial biofilm associated with implant material in clinical practice. Polym. J. 45, 137–152. doi: 10.1038/pj.2012.130

[ref58] Newton R. J. Jones S. E. Eiler A. McMahon K. D. Bertilsson S. (2011). A guide to the natural history of freshwater lake bacteria. Microbiol. Mol. Biol. Rev. 75, 14–49. doi: 10.1128/MMBR.00028-10, PMID: 21372319 PMC3063352

[ref59] Oh Y. J. Lee N. R. Jo W. Jung W. K. Lim J. S. (2009). Effects of substrates on biofilm formation observed by atomic force microscopy. Ultramicroscopy 109, 874–880. doi: 10.1016/j.ultramic.2009.03.042, PMID: 19394143

[ref60] Ortori C. A. Dubern J.-F. Chhabra S. R. Cámara M. Hardie K. Williams P.. (2011). Simultaneous quantitative profiling of N-acyl-L-homoserine lactone and 2-alkyl-4 (1H)-quinolone families of quorum-sensing signaling molecules using LC-MS/MS. Anal. Bioanal. Chem. 399, 839–850. doi: 10.1007/s00216-010-4341-0, PMID: 21046079

[ref61] Park S.-Y. Kang H.-O. Jang H.-S. Lee J.-K. Koo B.-T. Yum D.-Y. (2005). Identification of extracellular N-acylhomoserine lactone acylase from a Streptomyces sp. and its application to quorum quenching. Appl. Environ. Microbiol. 71, 2632–2641. doi: 10.1128/AEM.71.5.2632-2641.2005, PMID: 15870355 PMC1087586

[ref62] Pereira C. S. Thompson J. A. Xavier K. B. (2013). AI-2-mediated signalling in bacteria. FEMS Microbiol. Rev. 37, 156–181. doi: 10.1111/j.1574-6976.2012.00345.x, PMID: 22712853

[ref63] Pérez-Jiménez J. R. Young L. Y. Kerkhof L. J. (2001). Molecular characterization of sulfate-reducing bacteria in anaerobic hydrocarbon-degrading consortia and pure cultures using the dissimilatory sulfite reductase (dsrAB) genes. FEMS Microbiol. Ecol. 35, 145–150. doi: 10.1016/S0168-6496(00)00123-9, PMID: 11295453

[ref64] Persson T. Hansen T. H. Rasmussen T. B. Skindersø M. E. Givskov M. Nielsen J. (2005). Rational design and synthesis of new quorum-sensing inhibitors derived from acylated homoserine lactones and natural products from garlic. Org. Biomol. Chem. 3, 253–262. doi: 10.1039/B415761C, PMID: 15632967

[ref65] Qiu R. Zhao B. Liu J. Huang X. Li Q. Brewer E.. (2009). Sulfate reduction and copper precipitation by a Citrobacter sp. isolated from a mining area. J. Hazard. Mater. 164, 1310–1315. doi: 10.1016/j.jhazmat.2008.09.039, PMID: 18977087

[ref66] Rabe M. Verdes D. Seeger S. (2011). Understanding protein adsorption phenomena at solid surfaces. Adv. Colloid Interf. Sci. 162, 87–106. doi: 10.1016/j.cis.2010.12.007, PMID: 21295764

[ref67] Rodrigues C. M. Takita M. A. Coletta-Filho H. D. Olivato J. C. Caserta R. Machado M. A.. (2008). Copper resistance of biofilm cells of the plant pathogen Xylella fastidiosa. Appl. Microbiol. Biotechnol. 77, 1145–1157. doi: 10.1007/s00253-007-1232-1, PMID: 17992525

[ref68] Sani R. K. Peyton B. M. Brown L. T. (2001). Copper-induced inhibition of growth of <i>Desulfovibrio desulfuricans</i> G20: assessment of its toxicity and correlation with those of zinc and Lead. Appl. Environ. Microbiol. 67, 4765–4772. doi: 10.1128/AEM.67.10.4765-4772.2001, PMID: 11571183 PMC93230

[ref69] Sarker I. H. (2021). Machine learning: algorithms, real-world applications and research directions. SN Comput. Sci. 2:160. doi: 10.1007/s42979-021-00592-x, PMID: 33778771 PMC7983091

[ref70] Schäfer N. Schmidt B. C. Quéric N.-V. Röring B. Reitner J. (2015). Organic compounds and conditioning films within deep rock fractures of the Äspö hard rock laboratory. Geomicrobiol. J. 32, 231–242. doi: 10.1080/01490451.2014.911992

[ref71] Si M. Lang J. (2018). The roles of metallothioneins in carcinogenesis. J. Hematol. Oncol. 11:107. doi: 10.1186/s13045-018-0645-x, PMID: 30139373 PMC6108115

[ref72] Singh S. K. Roberts S. A. McDevitt S. F. Weichsel A. Wildner G. F. Grass G. B.. (2011). Crystal structures of multicopper oxidase CueO bound to Copper(I) and silver(I). J. Biol. Chem. 286, 37849–37857. doi: 10.1074/jbc.M111.293589, PMID: 21903583 PMC3199526

[ref73] Smith H. J. Schmit A. Foster R. Littman S. Kuypers M. M. M. Foreman C. M. (2016). Biofilms on glacial surfaces: hotspots for biological activity. NPJ Biofilms Microbiom. 2:16008. doi: 10.1038/npjbiofilms.2016.8, PMID: 28721245 PMC5515272

[ref74] Souza M. C. dos Santos L. S. Sousa L. P. Faria Y. V. Ramos J. N. Sabbadini P. S.. (2015). Biofilm formation and fibrinogen and fibronectin binding activities by Corynebacteriumpseudodiphtheriticum invasive strains. Antonie Van Leeuwenhoek 107, 1387–1399. doi: 10.1007/s10482-015-0433-3, PMID: 25828766

[ref75] Srivastava G. N. Malwe A. S. Sharma A. K. Shastri V. Hibare K. Sharma V. K. (2020). Molib: a machine learning based classification tool for the prediction of biofilm inhibitory molecules. Genomics 112, 2823–2832. doi: 10.1016/j.ygeno.2020.03.020, PMID: 32229287

[ref76] Stolle P. Hou B. Brüser T. (2016). The tat substrate CueO is transported in an incomplete folding state. J. Biol. Chem. 291, 13520–13528. doi: 10.1074/jbc.M116.729103, PMID: 27129241 PMC4919438

[ref77] Suzumoto Y. Dym O. Roviello G. N. Worek F. Sussman J. L. Manco G. (2020). Structural and functional characterization of new ssopox variant points to the dimer interface as a driver for the increase in promiscuous paraoxonase activity. Int. J. Mol. Sci. 21:1683. doi: 10.3390/ijms21051683, PMID: 32121487 PMC7084321

[ref78] Tripathi A. K. Saxena P. Thakur P. Rauniyar S. Samanta D. Gopalakrishnan V.. (2022). Transcriptomics and functional analysis of Copper stress response in the sulfate-reducing bacterium Desulfovibrio alaskensis G20. Int. J. Mol. Sci. 23, 1–25. doi: 10.3390/ijms23031396 PMC8836040 35163324

[ref79] Ueki T. Lovley D. R. (2022). Desulfovibrio vulgaris as a model microbe for the study of corrosion under sulfate-reducing conditions. mLife 1, 13–20. doi: 10.1002/mlf2.12018 PMC10989807 38818327

[ref80] Uroz S. Oger P. M. Chapelle E. Adeline M.-T. Faure D. Dessaux Y. (2008). A Rhodococcus qsdA-encoded enzyme defines a novel class of large-spectrum quorum-quenching lactonases. Appl. Environ. Microbiol. 74, 1357–1366. doi: 10.1128/AEM.02014-07, PMID: 18192419 PMC2258624

[ref81] Utari P. D. Vogel J. Quax W. J. (2017). Deciphering physiological functions of AHL quorum quenching acylases. Front. Microbiol. 8:1123. doi: 10.3389/fmicb.2017.01123, PMID: 28674525 PMC5474475

[ref82] Virpiranta H. Taskila S. Leiviskä T. Rämö J. Tanskanen J. (2019). Development of a process for microbial sulfate reduction in cold mining waters – cold acclimation of bacterial consortia from an Arctic mining district. Environ. Pollut. 252, 281–288. doi: 10.1016/j.envpol.2019.05.087, PMID: 31158656

[ref83] Warthmann R. Vasconcelos C. Sass H. McKenzie J. A. (2005). Desulfovibrio brasiliensis sp. nov., a moderate halophilic sulfate-reducing bacterium from Lagoa Vermelha (Brazil) mediating dolomite formation. Extremophiles 9, 255–261. doi: 10.1007/s00792-005-0441-8, PMID: 15856133

[ref84] Watson W. T. Minogue T. D. Val D. L. Von Bodman S. B. Churchill M. E. (2002). Structural basis and specificity of acyl-homoserine lactone signal production in bacterial quorum sensing. Mol. Cell 9, 685–694. doi: 10.1016/S1097-2765(02)00480-X, PMID: 11931774

[ref85] Whitehead K. A. Verran J. (2015). Formation, architecture and functionality of microbial biofilms in the food industry. Curr. Opin. Food Sci. 2, 84–91. doi: 10.1016/j.cofs.2015.02.003

[ref86] Wikieł A. J. Datsenko I. Vera M. Sand W. (2014). Impact of Desulfovibrio alaskensis biofilms on corrosion behaviour of carbon steel in marine environment. Bioelectrochemistry 97, 52–60. doi: 10.1016/j.bioelechem.2013.09.008, PMID: 24238898

[ref87] Wu S. Feng J. Liu C. Wu H. Qiu Z. Ge J.. (2022). Machine learning aided construction of the quorum sensing communication network for human gut microbiota. Nat. Commun. 13:3079. doi: 10.1038/s41467-022-30741-6, PMID: 35654892 PMC9163137

[ref88] Zampieri B. D. B. Nogueira E. W. de Oliveira A. Sanchez-Andrea I. Brucha G. (2022). Effects of metals on activity and community of sulfate-reducing bacterial enrichments and the discovery of a new heavy metal-resistant SRB from Santos port sediment (Sao Paulo, Brazil). Environ. Sci. Pollut. Res. Int. 29, 922–935. doi: 10.1007/s11356-021-15418-9, PMID: 34341933

[ref89] Zhao K. Tseng B. S. Beckerman B. Jin F. Gibiansky M. L. Harrison J. J.. (2013). Psl trails guide exploration and microcolony formation in Pseudomonas aeruginosa biofilms. Nature 497, 388–391. doi: 10.1038/nature12155, PMID: 23657259 PMC4109411

[ref90] Zobell C. E. (1943). The effect of solid surfaces upon bacterial activity. J. Bacteriol. 46, 39–56. doi: 10.1128/jb.46.1.39-56.1943, PMID: 16560677 PMC373789

