# Supplementary figures and images for: Molecular regulation of conditioning film formation and quorum quenching in sulfate reducing bacteria
Source: Front Microbiol. 2022 Oct 31;13:1008536. doi: 10.3389/fmicb.2022.1008536 (PMC9659907; doi:10.3389/fmicb.2022.1008536)

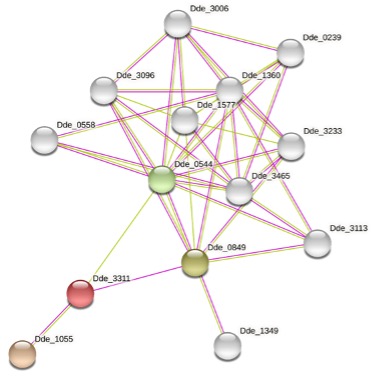

Supplement: Supplementary Figure 1 — Overview of molecular interactions and networking among all essential genes involved in the autoinducer release in Oleidesulfovibrio alaskensis G20. [file Image_1.JPEG]
